# POC1A acts as a promising prognostic biomarker associated with high tumor immune cell infiltration in gastric cancer

**DOI:** 10.18632/aging.103624

**Published:** 2020-10-14

**Authors:** Jun Lu, Xiao-Yan Huang, Yao-Hui Wang, Jian-Wei Xie, Jia-Bin Wang, Jian-Xian Lin, Qi-Yue Chen, Long-Long Cao, Ping Li, Chang-Ming Huang, Chao-Hui Zheng

**Affiliations:** 1 Department of Gastric Surgery, Fujian Medical University Union Hospital, Fuzhou, China; 2 Department of General Surgery, Fujian Medical University Union Hospital, Fuzhou, China; 3 Key Laboratory of Ministry of Education of Gastrointestinal Cancer, Fujian Medical University, Fuzhou, China

**Keywords:** gastric cancer, cell cycle, lymphocytes, immune infiltration, POC1A

## Abstract

The effect of POC1 centriolar protein A (POC1A) on gastric cancer (GC) has not been clearly defined. In this study, POC1A expression and clinical information in patients with GC were analyzed. Multiple databases were used to investigate the genes that were co-expressed with POC1A and genes whose changes co-occurred with genetic alternations of POC1A. Moreover, the TISIDB and TIMER databases were used to analyze immune infiltration. The GSE54129 GC dataset and LASSO regression model (tumor vs. normal) were employed, and 6 significant differentially expressed genes (LAMP5, CEBPB, ARMC9, PAOX, VMP1, POC1A) were identified. POC1A was selected for its high expression in adjacent tissues, which was confirmed with IHC. High POC1A expression was related to better overall and recurrence-free survival. GO and KEGG analyses demonstrated that POC1A may regulate the cell cycle, DNA replication and cell growth. Furthermore, POC1A was found to be correlated with immune infiltration levels in GC according to the TISIDB and TIMER databases. These findings indicate that POC1A acts as a tumor suppressor in GC by regulating the cell cycle and cell growth. In addition, POC1A preferentially regulates the immune infiltration of GC via several immune genes. However, the specific mechanism requires further study.

## INTRODUCTION

Gastric cancer (GC) is still one of the leading causes of cancer-related death worldwide [1]. Even though there are multiple surgical treatment options for GC, approximately 679,100 new cases and 498,000 new deaths pre-year demonstrated that the outcomes of GC are remain not optimistic [2, 3]. With further investigation was applied to the occurrence and progress of GC, the importance of underlying molecular mechanism attracted the attention of scientists. To improve the diagnosis and therapeutic response of GC, alternative biomarkers with higher accuracy and specificity are urgently needed.

With the advent of advanced techniques, a great number of proteins have been discovered to have an impact on GC. For instance, Wang et al. [4] found that Hsp90ab1, a member of the heat shock protein family, was found to be overexpressed and correlated with poor prognosis, proliferation and invasion in GC. Furthermore, MICAL-L2 was reported to promote the stability of EGFR to increase GC cell migration [5]. However, the known proteins that regulate GC development are still limited, which suggests that more innovative proteins should be discovered and further studied.

As a protein located in the centrosome, spindle apparatus and microtubule, POC1A (POC1 centriolar protein homolog A), which is also called WDR51A, is characterized by a conserved structure that consists of a 7-bladed β-propeller formed by an N-terminal WD40 domain and a C-terminal coiled-coil. In biological processes, POC1A plays an important role in the formation and steady state of centrioles, as well as in ciliogenesis. In addition, early steps of centriole duplication and later steps of centriole length control are also functions of POC1A [6, 7]. Many studies have shown that POC1A is associated with short stature, onychodysplasia, facial dysmorphism and hypotrichosis (SOFT) syndrome, which is related to abnormalities in cell mitosis [7, 8]. All these studies indicate that POC1A may play a critical role in cell proliferation by regulating mitosis. Furthermore, by RT-qPCR and immunohistochemistry staining, we discovered that POC1A, which is highly expressed in adjacent tissues of GC, acted as a tumor suppressor in GC. In recent decades, several studies have revealed that mitosis is an important factor in GC progression. Li et al. [9] discovered that KIF23 participates in the progression of cell mitosis and that its depletion inhibits cell proliferation in GC. Moreover, similar results were revealed by Zhang et al. [10]: PRKDC regulates the DNA damage response to affect proliferation induced by mitosis in GC. As demonstrated by several results above, we suspect that POC1A impacts the development of GC by regulating mitosis, which has never been reported. Therefore, our study aimed to investigate the regulation of POC1A on the progression of GC.

Immune infiltration was one of the several factors inducing GC. The chronic immune inflammation could lead to microbial infections, which may provide suitable environment for helicobacter pylori so that induce the development of GC [11]. Besides, as Chen et al. suggested, high destiny of T-bet+ tumor infiltrating lymphocytes (TILs) induced better survival in gastric cancer including overall survival (OS) and disease-free survival (DFS), which demonstrated that TILs could significantly impact the survival of gastric cancer [12]. Furthermore, Chen et al. indicated that over expression of the chemokine receptor CXCR3 could lead to better prognosis and higher immune infiltration levels including dendritic cells and CD8+ cells [13]. Several researches showed that the immune infiltration induced by immune signatures was significantly correlated with the prognosis of GC. Therefore, we focused on the immune infiltration in GC to investigate the correlated signatures regulating GC progress. In the progression of GC, endogenous genes and the exogenous tumor microenvironment (TME) are both significant factors [14–17], which indicates that investigating the relationship between biomarkers and the TME is meaningful. As components of the TME, tumor-infiltrating lymphocytes (TILs) and tumor-associated macrophages (TAMs) are also reported to have an important impact on the prognosis of GC [18, 19]. Therefore, investigation of the impact of different immune cells and immunotherapies on GC is extremely meaningful, and the immune microenvironment attracted our attention for exploring the function of POC1A.

## RESULTS

### Acquisition of differentially expressed genes

**Figure 1:**
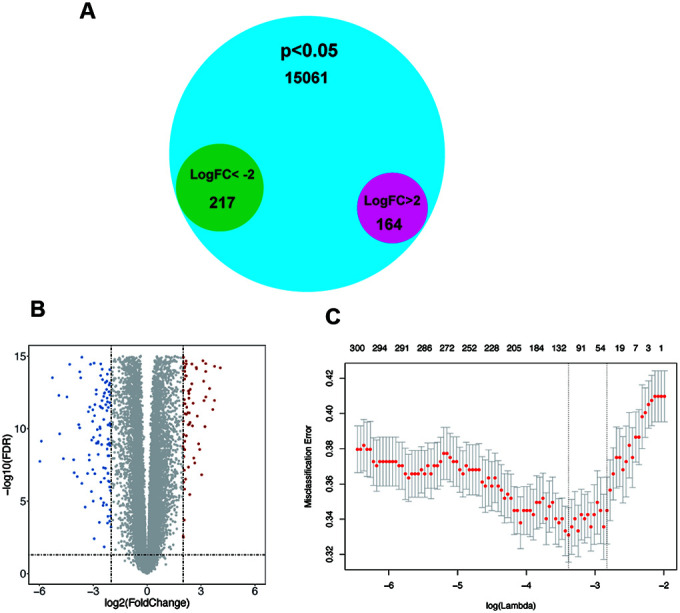
**The screening of significant genes.** (**A**–**B**) Venn plot and volcano plot demonstrating the expression of 164 upregulated genes and 217 downregulated genes in GSE54129 after differential expression analysis. (**C**) Six genes with the lowest misclassification error remained after LASSO regression analysis.

To determine significantly altered genes, a GEO dataset (GSE54129) was selected (Supplementary Figure 1A, 1B), and the Limma [20] R package was used for differential expression analysis (|logFC|>2, p<0.05). Thereby, we discovered 164 upregulated genes and 217 downregulated genes (Figure 1A, 1B). After using LASSO regression analysis, we established a statistical model by cross validation. As misclassification error was regarded as the minimized target parameter, we acquired 6 significantly differentially expressed genes, Lysosomal Associated Membrane Protein Family Member 5 (LAMP5), CCAAT Enhancer Binding Protein Beta (CEBPB), Armadillo Repeat Containing 9 (ARMC9), Polyamine Oxidase (PAOX), Vacuole Membrane Protein 1 (VMP1), POC1 centriolar protein A (POC1A) for further research (Figure 1C). In these 6 genes, only POC1A and PAOX were demonstrated to have down-regulated expression and other 4 genes were up-regulated.

### The expression of genes in GC tissues

**Figure 2:**
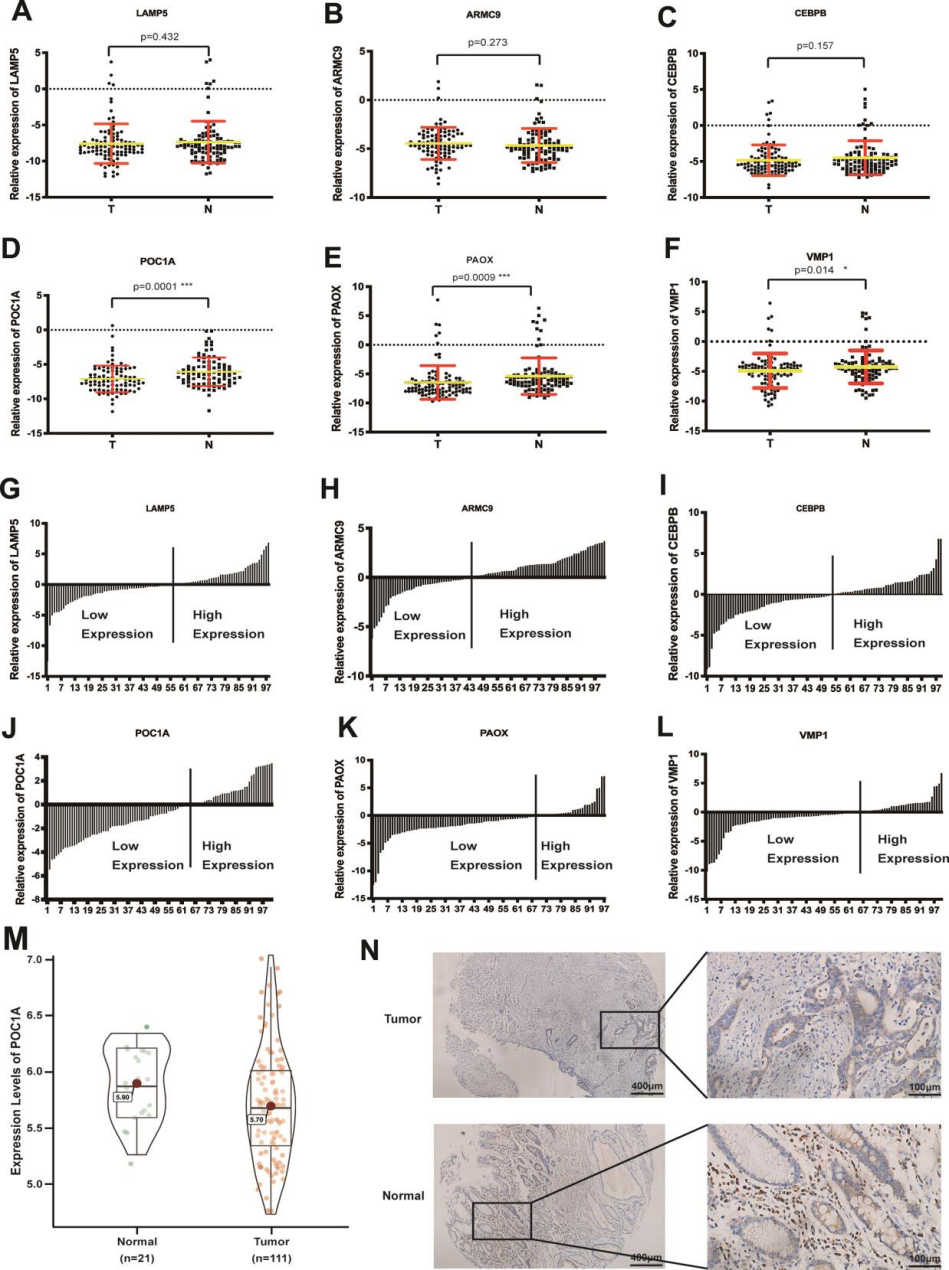
**POC1A was a significant anti-tumor gene in gastric cancer.** (**A**–**F**) Comparison of mRNA expression of 6 significant genes (LAMP5, CEBPB, ARMC9, POC1A, PAOX, VMP1) from LASSO regression analysis in tumor and adjacent tissues (n=101). (**G**–**I**). Ratio the mRNA expression of 6 genes comparing 2^(-CT)^ in tumor tissues with 2^(-CT)^ in matched normal tissues (n=101). (**M**) mRNA expression of POC1A in tumor (n=111) tissues was significantly higher than normal (n=21) tissues from GSE54129. (**N**) Intensity of immunohistochemistry staining of POC1A in normal tissue was higher than intensity in tumor tissue from the same patient.

To discover the most significant gene, RT-qPCR was used to evaluate the mRNA expression of 6 genes in 101 pairs of tumor and normal tissues. As scatter plots showed, POC1A had the most significant difference in expression in GC (p=0.0001) (Figure 2A–2F). In addition, as exhibited by the trend in Figure 2G–2L, POC1A was the most obviously downregulated gene. Furthermore, we analyzed POC1A expression in GSE54129 and further revealed significantly high POC1A mRNA expression in adjacent tissues (p<0.05) (Figure 2M). In addition, as shown in Figure 2N, POC1A protein expression was higher in normal tissues than in tumor tissues from the same patient. All the results above demonstrated that POC1A was not only corresponded with trend in bioinformatic analysis but also highly expressed in adjacent tissues compared with GC tissues.

### Correlation between POC1A expression and clinicopathological features

For further investigation, we analyzed the relationship between POC1A expression and several clinicopathological characteristics from 3 groups. In the RT-qPCR cohort, we identified a clinically significant cut-off point (0.02) for POC1A mRNA expression. Patients were divided into 2 groups based on high POC1A expression (-2.13±4.46, n=23) and low POC1A expression (-7.75±1.23, n=78). POC1A expression was inversely correlated with tumor size (p=0.043) and lymph node metastasis (p=0.001) in GC patients (Supplementary Table 2). To acquire more reliable data, patients from the immunohistochemistry group were analyzed, and multiple factors, including N stage (p=0.04), tumor size (p=0.05) and lymphatic or nervous invasion (p=0.036), were found to have a negative correlation with POC1A protein expression (Supplementary Table 3). Moreover, with the same method applied to define POC1A high/low expression, the results of the correlation analysis in GSE84433 demonstrated that POC1A mRNA expression was negatively correlated with T stage (p=0.002) and lymph node status (p=0.008) (Supplementary Table 4).

### POC1A expression is correlated with lymphatic metastasis and GC

**Figure 3:**
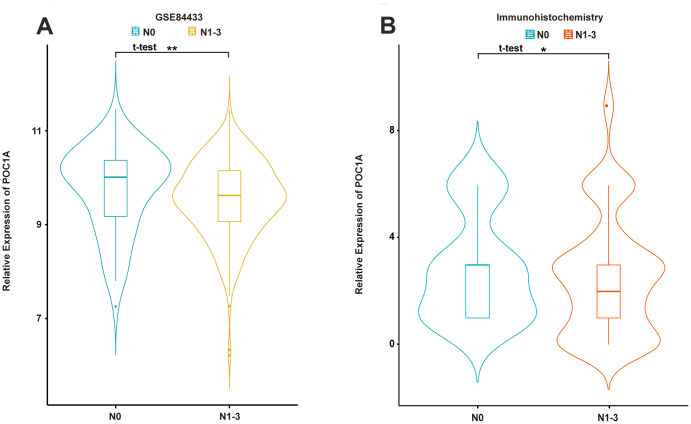
**POC1A was significantly correlated with lymph node metastasis.** (**A**, **B**) Expression of POC1A in patients with/without lymph node metastasis from GSE84433 analysis (n=357) and immunohistochemistry analysis (n=91).

Considering the results above, lymph node status was suggested to have a significant correlation with POC1A expression among the 3 groups, which demonstrated that POC1A could be regarded as a meaningful factor impacting lymph node metastasis in GC. Therefore, the GSE84433 dataset was selected, and patients were divided according to the presence of lymph node metastasis (N0, N1-3). The results suggested that patients in the N0 group, who had no lymph node metastasis, had higher POC1A mRNA expression than patients in the N1-3 group (Figure 3A). Furthermore, similar results were acquired from immunohistochemistry staining of POC1A protein (Figure 3B). High POC1A expression in patients without lymph node metastasis indicated that POC1A may act as a suppressor of lymph node metastasis in GC.

### Prognostic potential of POC1A in GC

**Figure 4:**
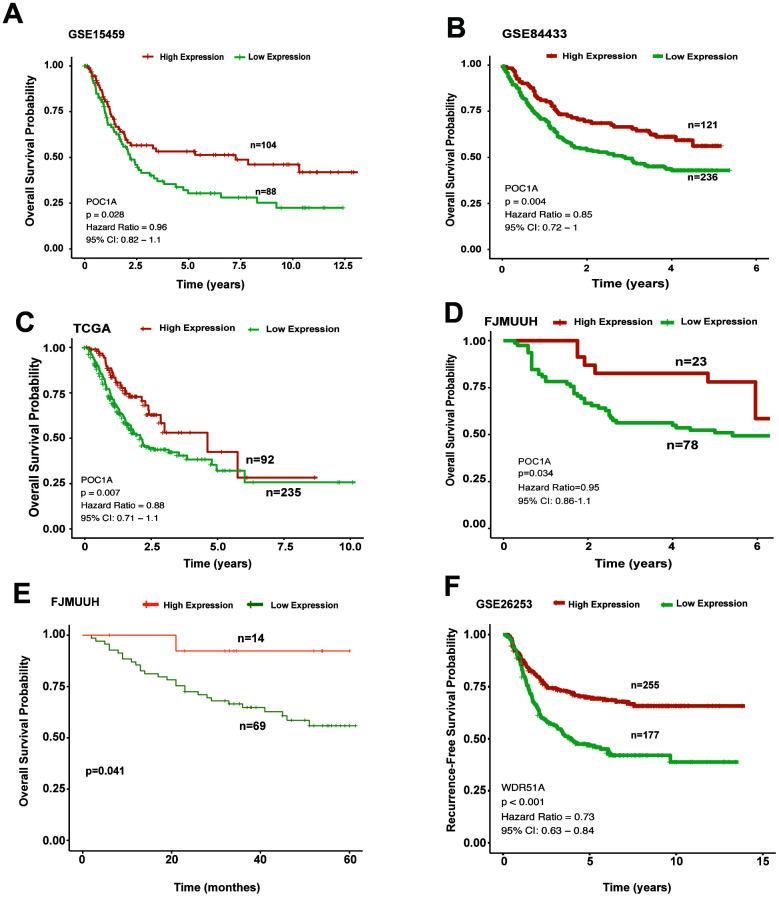
**High POC1A expression significantly induced better survival outcomes.** (**A**–**C**) Survival curves of OS in 3 cohorts (GSE15459, GSE84433, STAD of TCGA) (cut-off: 7.97,n=192, p=0.028; cut-off: 9.3, n=357, p=0.004; cut-off: 9.45, n=327, p=0.007) demonstrated that high POC1A mRNA expression led to better overall survival in GC. (**D**) High POC1A expression was significantly correlated with better overall survival (OS) prognosis in the RT-qPCR cohort (n=101, p=0.034). (**E**) Low POC1A protein expression led to poor OS prognosis in the immunohistochemistry cohort (n=83). (**F**) RFS curves of patients from GSE26253 (cut-off: 7.67, n= 432).

To understand the role of POC1A in survival outcomes, we analyzed the impact of POC1A on prognosis in GC. In the overall survival analysis of multiple cohorts (GSE15459, GSE84433 and TCGA-STAD) (p=0.028, p=0.004, p=0.007), patients in the high POC1A expression group tended to have better overall survival than those in the low POC1A expression group (Figure 4A–4C), as did patients in our department (FJMUUH, Figure 4D). In addition, Kaplan-Meier analysis of immunohistochemistry results, which included 83 patients with OS data, revealed that high POC1A protein expression (IHS>=3, n=14) was related to better overall survival than low POC1A expression in GC (IHS<3, n=69) (Figure 4E).

Furthermore, the same method was adopted for analyzing recurrence-free survival (RFS). We applied another GEO dataset (GSE26253) for further investigation and found that degradation of POC1A was significantly related to poor RFS in GC (Figure 4F). Thus, all survival analyses indicated that POC1A acts as a tumor suppressor in GC.

### Genes coexpressed with POC1A in STAD

**Figure 5:**
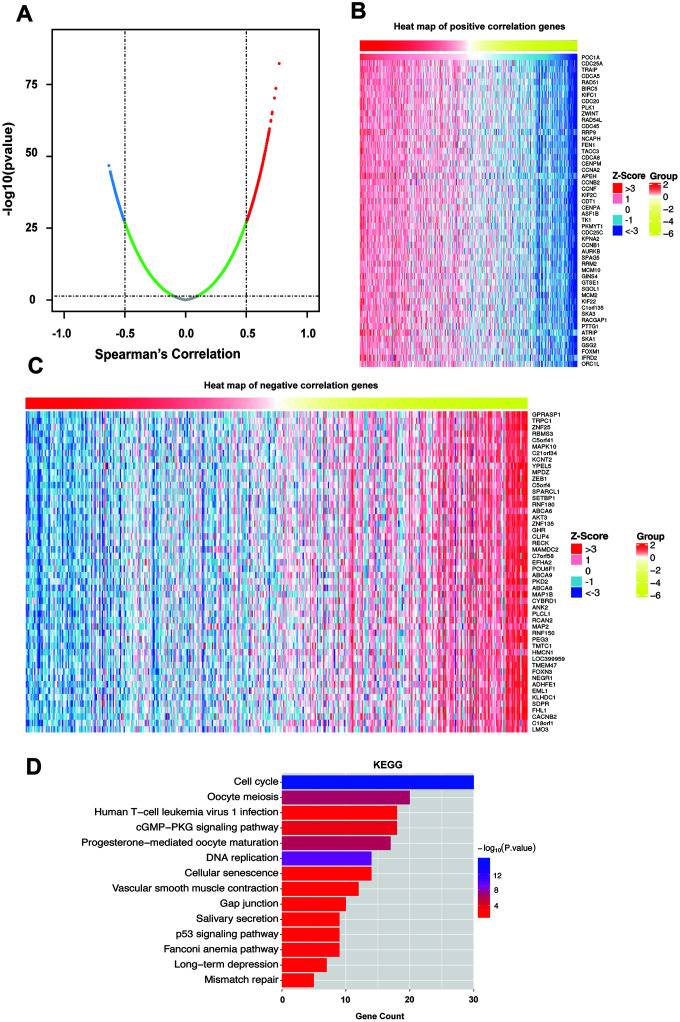
**Coexpressed genes of POC1A.** (**A**) Strongly coexpressed genes of POC1A identified by Spearman’s test in GC. (**B**–**C**) Heat maps exhibited the top 50 genes that have positive and negative correlations with POC1A in GC. Red represents genes with strong positive correlation, and blue indicates genes with strong negative correlation. (**D**) KEGG pathway enrichment of genes coexpressed with POC1A.

To determine the biological role of POC1A, we selected LinkedOmics to examine genes coexpressed with POC1A in STAD. As the results in Figure 5A suggest, 261 genes (red dots) were demonstrated to have a significant strong positive correlation with POC1A, whereas 355 genes (blue dots) had a significant negative correlation with POC1A (false discovery rate, FDR<0.01, |Spearman’s correlation|>0.5). The top 50 significant genes that were positively and negatively correlated with POC1A are shown as heat maps (Figure 5B, 5C). Significant Gene Ontology (GO) and Kyoto Encyclopedia of Genes and Genomes (KEGG) analyses were performed (Figure 5D, Supplementary Figure 1C–1E). The KEGG results showed that the cell cycle, DNA replication, p53 signaling pathway, etc. (Figure 5D and Supplementary Table 5) were suggested to be enriched pathways. The results of GO analysis indicated that chromosome segregation, DNA replication, cell cycle DNA replication, etc. (Supplementary Figure 1C–1E and Supplementary Table 6) were involved. Furthermore, we used the STRING database to analyze these strongly coexpressed genes of POC1A and acquired the protein-protein interaction (PPI) network (Supplementary Figure 1F). All these results demonstrated that POC1A may affect the cell cycle and DNA replication as a part of the centrosome.

### Changes in gene expression cooccurring with POC1A deletion in STAD

**Figure 6:**
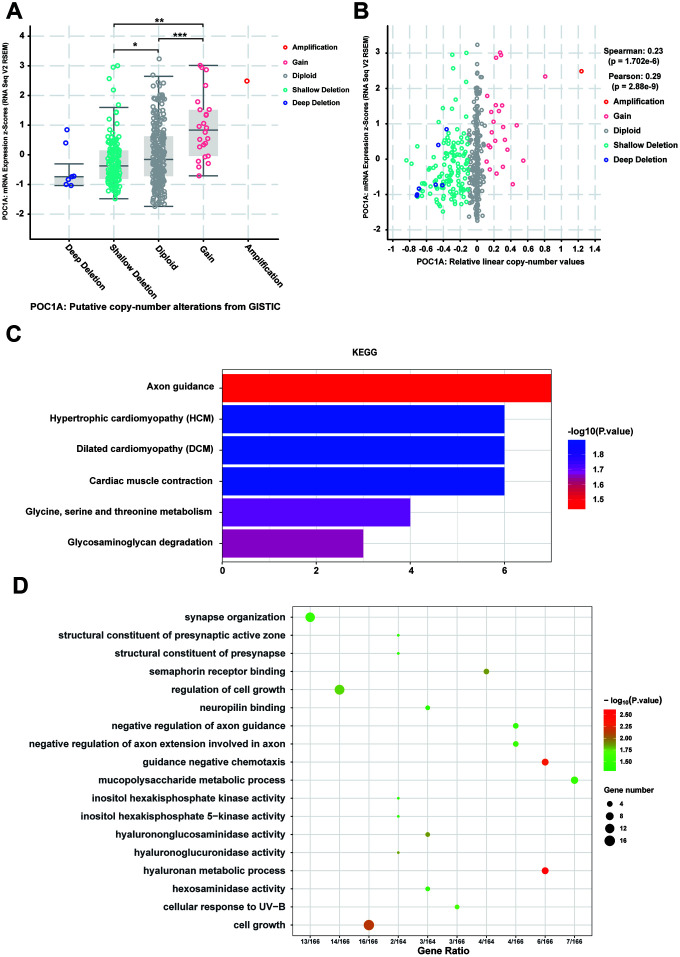
**CNV co-occurrence profiles of POC1A in STAD.** (**A**–**B**) POC1A deletion was significantly correlated with low POC1A expression (Spearman: r=0.23, p=8.129e-6; Pearson: r=0.29, p=8.53e-9), which indicated that POC1A deletion may act as a significant pathogenic factor. (**C**–**D**) GO and KEGG analysis of POC1A co-occurrence genes (p<0.05).

Considering that genomic alterations of POC1A are pathogenetic, copy number alteration (CNA) of POC1A attracted our attention. With the cBioPortal database, we discovered that POC1A deletion was significantly correlated with its decreased mRNA expression in STAD (Figure 6A, 6B). Thus, we further examined genes with changes occurring with POC1A deletion in STAD, revealing 212 genes (Supplementary Table 7). Then, we performed KEGG pathway analysis of these genes and found that glycine, serine and threonine metabolism and some other pathways were enriched (Figure 6C, Supplementary Table 8). In addition, analysis of GO enrichment demonstrated that these genes primarily induce cell growth and regulate of cell growth (Figure 6D, Supplementary Table 9), which corresponded to the result that POC1A expression was correlated with tumor size (Supplementary Tables 2, 3).

### The impact of POC1A in tumor-infiltrating lymphocytes

**Figure 7:**
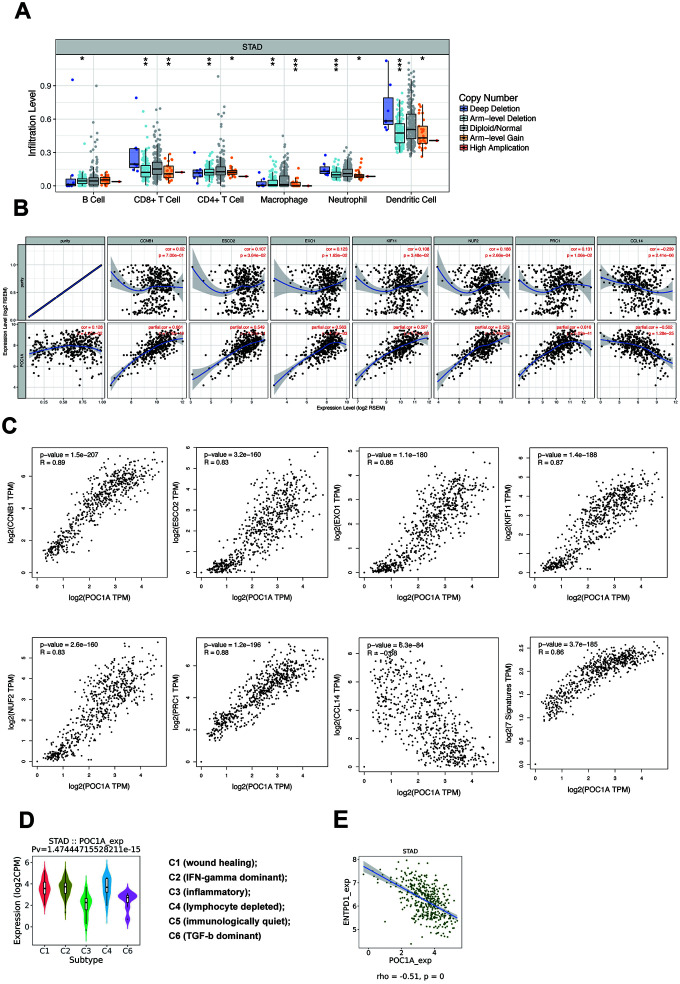
**POC1A could impact immune infiltration significantly.** (**A**) Copy number alteration (CNA) of POC1A was significantly correlated with immune infiltration levels of several immune cell types in GC. Deletion was found to have a highly reliable significant correlation with the infiltration level in neutrophils and dendritic cells (p<0.001), and arm-level gain was related to the infiltration level of macrophages with high reliability (p<0.001). (**B**) In the TIMER database, scatterplots of strong (|r|>0.5) and significant (p<0.001) correlations between POC1A expression and 7 immune genes (CCNB1, ESCO2, EXO1, KIF11, NUF2, PRC1, CCL14) after adjusting for purity. (**C**) In the GEPIA database, scatterplots of correlations between POC1A expression and 7 immune genes from TIMER. (**D**) Expression of POC1A in different immune subtypes of GC. (**E**) ENTPD1 had a strong significant correlation with POC1A (r=-0.51, p<0.001).

Tumor-infiltrating lymphocytes (TILs) are an important factor affecting lymph node status in several cancers [21]. As POC1A can impact lymph node metastasis, immune infiltration attracted our attention. According to the TIMER database, multiple types of copy number alterations (CNAs) of POC1A, especially the POC1A deletion, had significant correlations with the infiltration levels of several immune cells in stomach adenocarcinoma (Figure 7A and Supplementary Table 10). The results above indicated that POC1A deletion in STAD induced low immune infiltration of multiple immune cells. However, the amplification of POC1A also induced the lower immune infiltration level, which need further discussion. Then, we selected and downloaded multiple immune genes from the TIMER database to study immune infiltrating marker sets and performed correlation analysis for further investigation (Supplementary Table 11). As shown in Figure 7B, after adjusting for purity, 7 genes (CCNB1, ESCO2, EXO1, KIF11, NUF2, PRC1, CCL14) were suggested to have a strong and significant relationship with POC1A expression (|r|>0.5, p<0.001). We further verified the markers above through GEPIA, whose results were similar (Figure 7C). Among the 7 genes, 6 genes (CCNB1, ESCO2, EXO1, KIF11, NUF2, PRC1) were expressed in CD4+ T cells, and only CCL14 was featured in macrophages, which indicated that CD4+ T cells may play an important role in the impact of POC1A on immune infiltration.

### The potential of POC1A in immune therapy

While multiple immune markers were found to be correlated with POC1A expression, enough attention has not been paid to the most significantly related genes. Therefore, we further employed the TISIDB database [22] to further understand POC1A and other biomarkers in immune therapy. By dividing GC into 6 groups (wound healing, IFN-gamma dominant, inflammatory, lymphocyte depleted, immunologically quiet, TGF-b dominant) [23], we found that POC1A expression was the highest in the IFN-gamma dominant subtype and the lowest in the inflammatory subtype (Figure 7D). Furthermore, we analyzed the correlation through TISIDB and stetted filter standard (|r|>0.1 and p<0.05). In total, 12 MHC molecules, 16 immunoinhibitors and 30 immunostimulators that had a significant correlation with POC1A expression in STAD were discovered (Supplementary Figures 2–4). After defining the thresholds as |r|>0.5 and p<0.01 to acquire effective immunomodulators, only ENTPD1, an MHC molecule, was found to have a strong correlation with POC1A expression (Figure 7E). This demonstrated that POC1A has the potential to impact the immune therapy induced by ENTPD1 in STAD. All these results indicated that POC1A has the potential to regulate immune infiltration and the response to immunotherapy, but these hypotheses need to be verified by further studies.

## DISCUSSION

As the core component of the centrosome, POC1A is regarded as a factor that regulates the cell cycle; mutation of POC1A can lead to abnormal mitotic mechanics, multipolar spindles and impaired ciliogenesis which can induce short stature, onychodysplasia, facial dysmorphism, and hypotrichosis (SOFT) [24, 25]. Many articles have reported the function of the centrosome in cancer, and centrosome regulation is a significant factor in the development of cancer processes. Arunabha Bose et al. [26] revealed that centrosome amplification is a significant characteristic in multiple cancer types and contributes to both tumor initiation and tumor progression. As Wang et al. [27] discovered, SCL/TAL1 interrupting locus (STIL) induced tumor progression by promoting centriolar replication and cell cycle progression by regulating the mitotic centrosome. Therefore, we wandered whether POC1A, as a centrosome regulator, could act as a sensitive biomarker in GC. Herein, we investigated POC1A expression and its effects on prognosis in GC. To our knowledge, this is the first study to highlight POC1A not only as a tumor suppressor but also as an immune-infiltrating protein in GC.

First, we identified several differentially expressed genes from a GEO dataset (GSE54129) by applying the Limma R package, and LASSO regression analysis revealed 6 significant genes. Then, RT-qPCR was applied to analyze these genes, and POC1A was selected for its significantly high expression in adjacent tissues compared to cancer tissues. As the results of RT-qPCR, immunohistochemistry and several GEO datasets show, not only the mRNA expression but also the protein expression of POC1A is higher in cancer-adjacent tissues than in tumor tissues. In addition, we also found that POC1A expression was significantly correlated with tumor size and lymphatic metastasis in 3 cohorts (Supplementary Table 2–4). Moreover, after Kaplan-Meier survival analysis, high overall survival and high recurrence-free survival were shown to correlate with high POC1A expression in GC, which indicates that POC1A produces an antitumor effect in GC. As POC1A could impact prognosis of GC patients, we further analyzed POC1A in different stages. However, there were no significant difference among different stages in STAD. Therefore, we further employed TISIDB to investigate the correlation between POC1A and different stages, which demonstrated that POC1A expression was decreasing with the development of TNM stages in STAD and the correlation was significant (rho=-0.1, p=0.02) (Supplementary Fig5 a). Thus, we hold the view that POC1A has the potential to be a diagnostic and prognostic marker.

In addition, to further investigate the biological function of POC1A in the development of GC, we discovered multiple genes coexpression with POC1A genes, which were further subjected to GO and KEGG enrichment analyses. Our results show that POC1A may produce effects through these factors to regulate the cell cycle and DNA replication. Then, a protein-protein interaction network of these coexpressed genes was established to provide a basis for investigating the mechanism underlying the antitumor effect of POC1A.

However, analysis of coexpressed genes cannot explain the impact of POC1A on tumor volume and lymphocyte metastasis. Therefore, we focused on genomic alterations. It has been reported that copy number alteration (CNA) can lead to abnormal gene expression, which induces several genetic diseases. Therefore, as the majority of studies of POC1A reported its gene alteration could lead to genetic disease [7, 8, 24, 25], we verified the positive relationship between POC1A CNA and POC1A expression and investigated changes in gene expression that occurred with POC1A CNA in STAD. A total of 212 genes had expression changes that cooccurred with POC1A deletion. The GO and KEGG analyses of these genes suggested that POC1A deletion may induce cell growth, which is consistent with our results that POC1A expression is significantly correlated with tumor size.

Another aspect of our study is that POC1A is correlated with immune infiltration, which is a significant factor in the microenvironment of GC. As many articles have reported, a great deal of immune cells, including lymphocytes and macrophages, induce an immune microenvironment of GC, which consequently affects the prognosis of GC patients [18, 28–30]. In addition, the association between increased expression of POC1A and the prognosis of patients who have GC with lymph node metastasis indicates that POC1A can be regarded as a significant factor in tumor metastatic progression. It has also been reported that lymphocyte metastasis may also be impacted by tumor-infiltrating lymphocytes [21]. Thus, we analyzed the relationship between POC1A genomic alterations and immune infiltration in GC. However, as POC1A has no significant correlations with tumor purity, we considered that POC1A mutation may lead to genetic diseases [8, 25, 27], so we wanted to determine whether the genomic alteration of POC1A was correlated with immune infiltration levels. With the TIMER database, our results first demonstrated that deletion of POC1A copy number alteration (CNA) had a meaningful correlation with the immune infiltration level of multiple immune cell types, which provides a new foundation and direction for further research of POC1A in GC. We employed the cBioPortal database to measure POC1A mRNA expression in different CNA types and revealed that the CNA of POC1A is positively related to POC1A mRNA expression. As shown above, POC1A deletion significantly reduced immune infiltration, which indicated that the anticancer effect of POC1A in GC is regulated by immune infiltration of the microenvironment via genomic alteration. Besides, we found that amplification also induced the lower immune infiltration level. Through the further investigation, we found that the correlation of POC1A among different subtypes of the same immune cell was different. For example, POC1A was positively correlated with macrophage M0 and M1 in multiple databases while it was negatively correlated with macrophage M2 (Supplementary Figure 5B–5F), which may induce the negative correlation between POC1A expression and macrophage. Therefore, even though the alteration of copy number could significantly regulate the POC1A expression, the infiltration levels of several cells in Figure 7A may not be corresponding.

Immune signatures are significant biomarkers that predict prognosis in malignant cancer. Further study of immunogenomics will enhance immunotherapy treatment [31]. Long et al. [32] demonstrated that EXO1 is an immune gene that significantly affects prognosis in HCC. Dai et al. [33] found that Epstein–Barr virus (EBV) in GC can lead to PD-L1 positivity and high TIL density, which reduce survival benefits in GC patients. Therefore, to provide more support for our research on immune infiltration, we decided to find the association between POC1A and multiple classic immune biomarkers. Ultimately, 7 significant signatures (CCNB1, ESCO2, EXO1, KIF11, NUF2, PRC1, CCL14) were selected for their strong correlation. These results indicated that POC1A has the potential to act as a significant biomarker to impact the prognosis of GC by regulating immune infiltration levels via these immune signatures. Moreover, as POC1A was reported to have a significant relationship with the lack of response to atezolizumab in urothelial cancer [34], we wondered whether POC1A could function in the response to immune infiltration therapy and focused our attention on the association between POC1A expression and immunomodulator expression. Data from Charoentong et al. [35] and the TISIDB were used to investigate the correlation between immunomodulators and POC1A, and 58 significantly correlated immune immunomodulators were discovered. After filtering these signatures, only ENTPD1 exhibited a strong correlation, which provides a new direction of POC1A in GC immunotherapy. In previous studies, ENTPD1 (also known as CD39) was reported to act as an immune signature which was presented on the surface of several immune cells, including activated cells, T cells, regulatory T cells, endothelial cells and lymphocytes [36–39]. As an immune signature, ENTPD1 was reported to regulate immune infiltration. In the study of Rissiek et al. [40], ENTPD1(+) Tregs could suppress T cells proliferation and inflammatory cytokine production. Furthermore, as Cai et al. [41] reported, high level ENTPD1 may induce poor prognosis in gastric cancer and high CD39+/CD8+ could lead to poor overall survival, which was corresponding to the negative correlation between POC1A and ENTPD1. Therefore, we speculate that POC1A may impact outcome of GC patients though immune regulation which may be induced by ENTPD1.

However, our study has limitations. The reliability of the analyses of molecular mechanism and immune infiltration levels, especially the correlation between immune signatures and POC1A, is not supported by experiments in vivo and in vitro. More experiments for verifying the functions of these small molecules can benefit the in-depth understanding of POC1A in GC and encourage progress in diagnosis and therapy. Therefore, the molecular mechanism of immune infiltration still needs further study.

## CONCLUSIONS

In summary, we discovered POC1A as a tumor suppressor and found its potential role in affecting the prognosis of GC. In addition, coexpressed genes and genes with changes concurrent with POC1A CNA were predicted to regulate the cell cycle, DNA replication and cell growth in GC. Moreover, CD4+ T cell infiltration and an immunostimulator were found to have a significant strong correlation with POC1A, which provides a new direction for investigating this new biomarker in GC.

## MATERIALS AND METHODS

### Patients and specimens

The study employed 2 independent groups of fresh tumor and adjacent tissues from patients with GC who underwent surgery at Fujian Medical University Union Hospital (FJMUUH, Fuzhou, China). The POC1A mRNA expression group included 101 patients who underwent surgery between October 2013 and December 2018. Another group used for POC1A immunohistochemistry included 91 patients with gastrectomies between March 2013 and May 2015. None of the GC patients had received any preoperative chemotherapy or radiotherapy. All clinical pathological information, including sex, age, tumor size, tumor site, differentiation grade, and TNM stage (AJCC 7^th^ edition) [42] were reassessed independently by 2 pathologists. The time from surgery to death or the last follow-up was regarded as overall survival (OS). Recurrence-free survival (RFS) was defined as the time from surgery to recurrence. All specimens were stored in liquid nitrogen at -80 °C. The use of human samples was approved by the Institutional Review Board of FJMUUH. Written informed consent was obtained from our patients to acknowledge the use of their resected tissues for research purposes.

### Quantitative reverse transcriptase PCR

We selected 101 pairs of fresh tumor and adjacent tissues to measure POC1A mRNA expression and used TRIzol reagent for extracting total RNA, which was further transcribed to cDNA through reverse transcription. RT-qPCR was performed with SYBR qPCR mix (Takara Bio Inc) in a 7500 real-time PCR System (Thermo Fisher Scientific). GAPDH, whose primers were 5’-CTCGCTTCGGCAGCACA-3’ (forward) and 5’-AACGCTTCACGAATTTGCGT-3’ (reverse), was regarded as an endogenous reference for normalization. Other primer information is shown in Supplementary Table 1. Finally, we used the 2-ΔCt formula to represent the expression of the genes above.

### Tissue microarray and immunochemistry tissue

Tissues from 91 patients were fixed with formalin. After embedding with paraffin, tissues were made into a tissue microarray. Then, we used xylene for deparaffinization, and a grade alcohol series was used for hydration. After citric acid solution was applied for antigen retrieval, we used 3% H2O2 to inhibit endogenous peroxidase. Then, we incubated sections with 10% normal goat serum for 30 minutes. Primary antibody against POC1A (diluted 1:100, Sigma) was applied overnight. Subsequently, goat anti-rabbit IgG (1:1000, Boster Biological Technology) was used as the secondary antibody for incubation. After staining, 2 pathologists evaluated the results of each specimen according to the intensity of expression. The immunohistochemical score (IHS) was qualified by calculating the product of a 4-value intensity score (0, 1, 2, 3) and a 4-value percentage score, which included scores of 0 (0-5%), 1 (6-25%), 2 (51-75%), and 3 (76-100%). Afterwards, IHS<3 was defined as low expression, and high POC1A expression ranged from 3-9 [43].

### Immune infiltration database

We used the TIMER database (https://cistrome.shinyapps.io/timer/) to analyze the correlation between POC1A copy number alteration (CNA) and immune infiltration level. Moreover, the relationship between POC1A expression and immune infiltrating genes was also revealed. Furthermore, we employed the TISIDB database to evaluate whether POC1A was related to immune infiltration therapy and subtypes in stomach adenocarcinoma (STAD).

### TCGA and GEO database analysis

GSE54129 was selected from the Gene Expression Omnibus (GEO, http://www.ncbi.nlm.nih.gov/geo/) database, which was used to find significantly differentially expressed genes. Moreover, GAE8433, GSE15459 and GSE26253 were separately used for overall survival and recurrence-free survival analysis in terms of POC1A, whose expression between tumor and normal tissues was verified by GSE54129. In addition, we also acquired data from The Cancer Genome Atlas (TCGA) to analyze the prognosis of POC1A in STAD.

### GEPIA database analysis

As an online database, Gene Expression Profiling Interactive Analysis (GEPIA) (http://gepia.cancer-pku.cn/) is a database that involves tumor and normal samples from TCGA and GTEx [33]. We analyzed the relationship between POC1A and several immune signatures through the database.

### cBioPortal database analysis

cBioPortal (https://www.cbioportal.org/) [31, 44] is an open-access database that was sourced from TCGA and 225 cancer studies. We employed it to acquire copy number alteration (CNA) and cooccurrence genes of POC1A in STAD. We then performed GO and KEGG analysis of these cooccurrence genes through the “clusterProfiler” package in R [45], which evaluated cellular components (CCs), biological processes (BPs), molecular functions (MFs), and KEGG pathways.

### LinkedOmics database analysis

LinkedOmics (http://www.linkedomics.org/login.php) [46] is a cancer-associated database for analyzing TCGA. We used it to discover genes in TCGA STAD that were correlated with POC1A according to Spearman correlation and selected *LinkFinder* in LinkedOmics to establish a heatmap plot for the coexpressed genes. After acquiring strongly correlated coexpressed genes, we performed GO and KEGG analyses of these genes with the clusterProfiler R package [45].

### STRING database analysis

To investigate the interaction among the co-occurrence altered genes, we used the STRING v11 database [47] (https://string-db.org/) to analyze the relationship among 212 genes and construct a PPI network.

### Statistical analysis

As all Kaplan-Meier plots had P-values, survival curves were generated by the survival R package. In addition, the correlation between POC1A expression and clinical pathological characteristics of patients from RT-qPCR, immunohistochemistry and GSE84433 was analyzed with SPSS version 25.0 software. Furthermore, determination of the best cut-off point and LASSO regression analysis were separately performed with the survivalROC [48] and glmnet [49] R packages.

### Ethics approval

The study was approved by Fujian Medical University Union Hospital, China.

## Supplementary Material

Supplementary Figures

Supplementary Tables 1, 2, 3, 4 and 5

Supplementary Table 6

Supplementary File 7

Supplementary Tables 8, 9 and 10

Supplementary Table 11
