# Supplementary material for: POC1A acts as a promising prognostic biomarker associated with high tumor immune cell infiltration in gastric cancer
Source: Aging (Albany NY). 2020 Oct 14;12(19):18982–9011. doi: 10.18632/aging.103624 (PMC7732308; doi:10.18632/aging.103624)
Supplement: Supplementary Figures [file aging-12-103624-s001..pdf]

SUPPLEMENTARY FIGURES

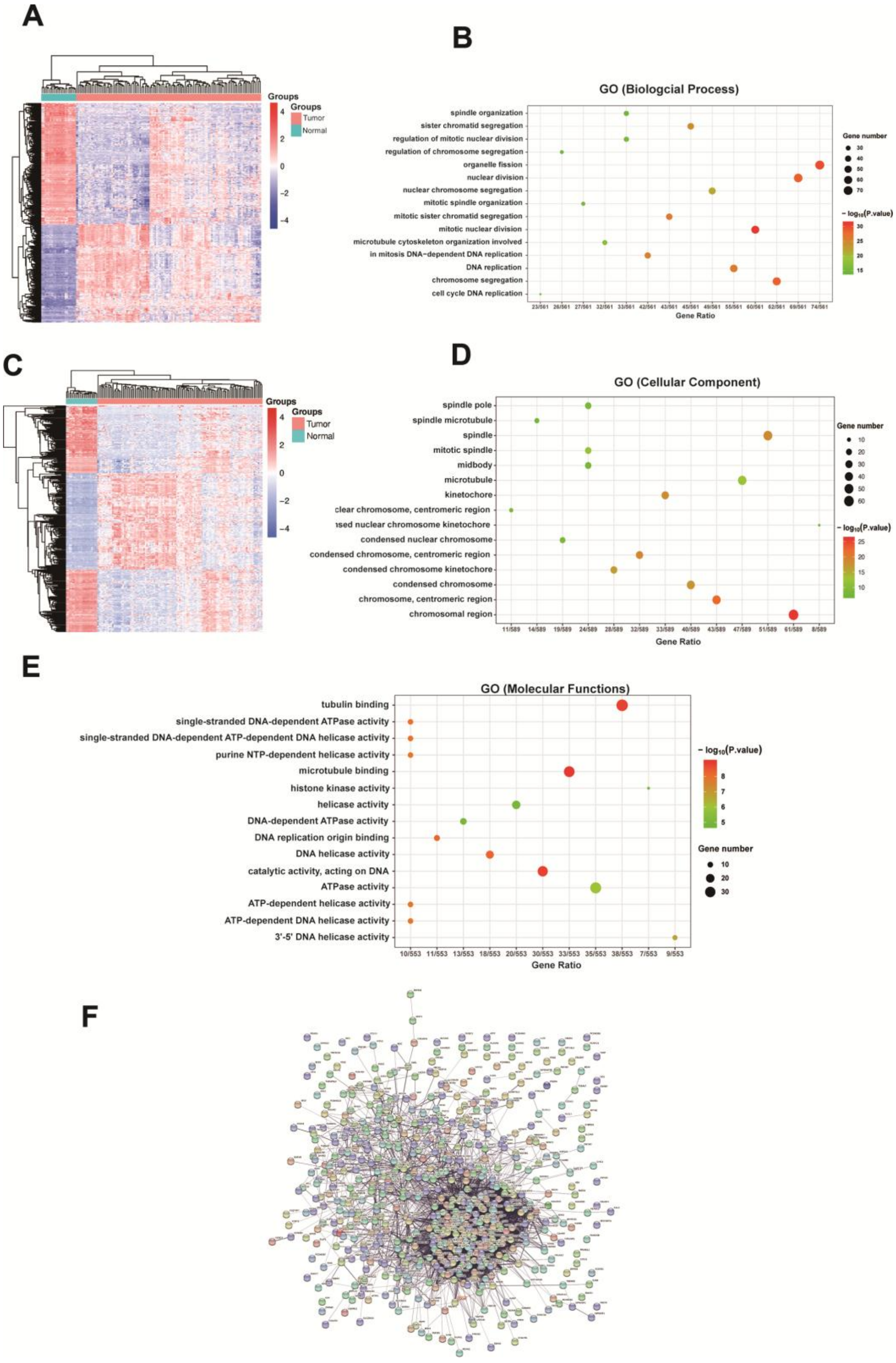

Supplementary Figure 1. The bioinformatic analysis of POC1A. (**A**, **B**) Heat map showing the expression of all genes and differently expressed genes in GSE54129; (**C–E**) GO enrichment analysis of POC1A co-expression genes; (**F**) Protein-protein interaction (PPI) network of genes strongly coexpressed with POC1A.

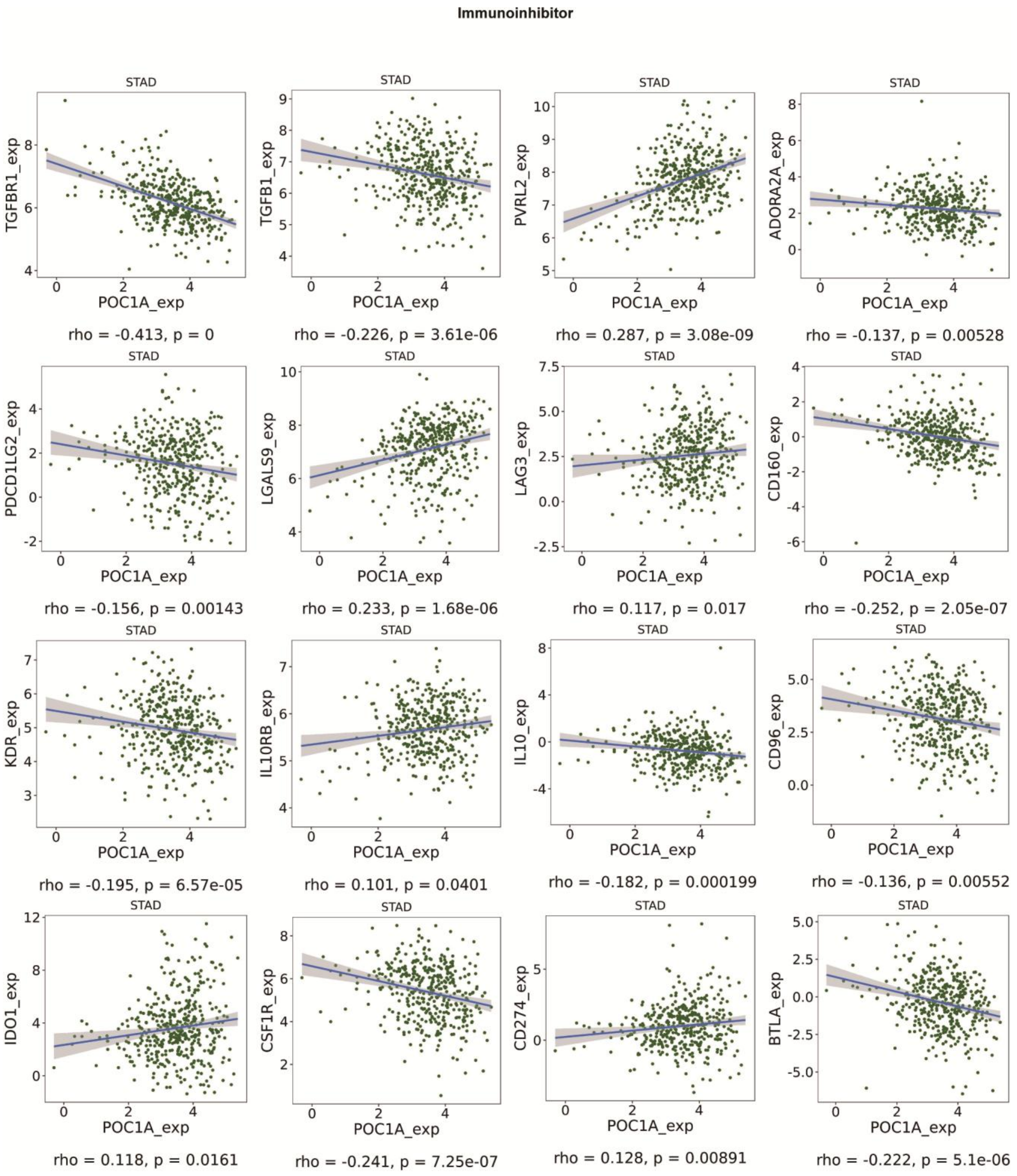

Supplementary Figure 2. Significant correlation between POC1A and several immunoinhibitors.

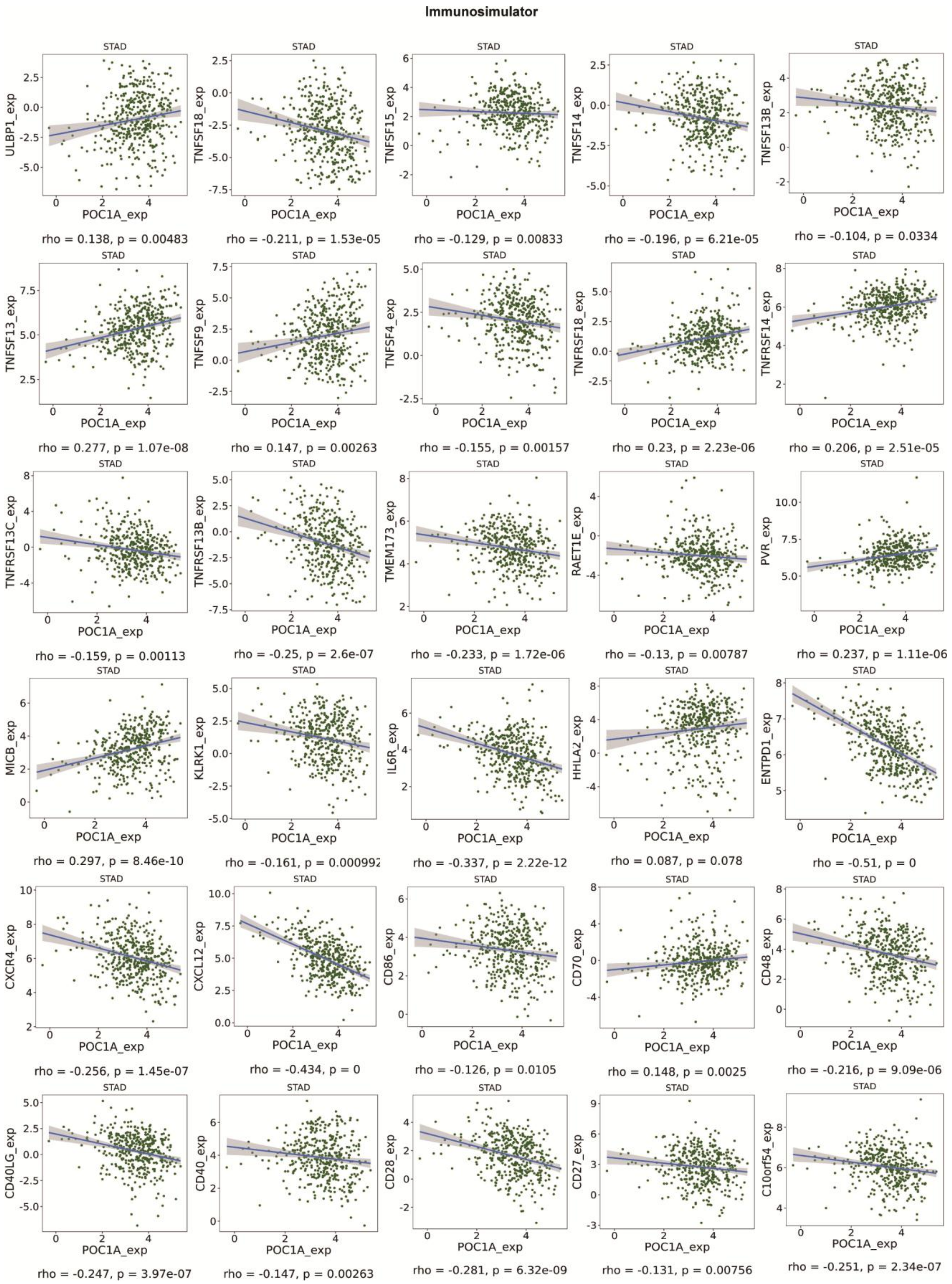

Supplementary Figure 3. Significant correlation between POC1A and several immunostimulators.

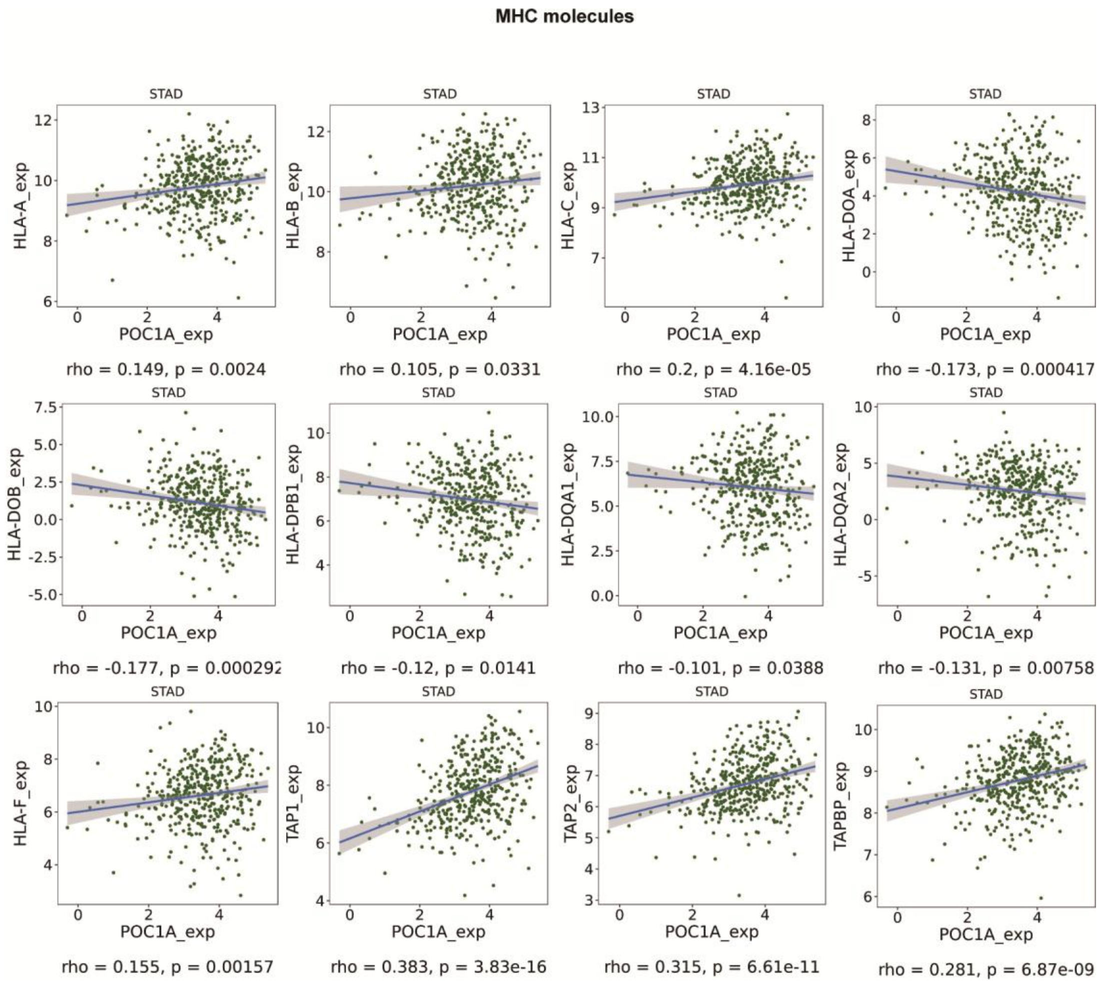

Supplementary Figure 4. Significant correlation between POC1A and several MHC molecular.

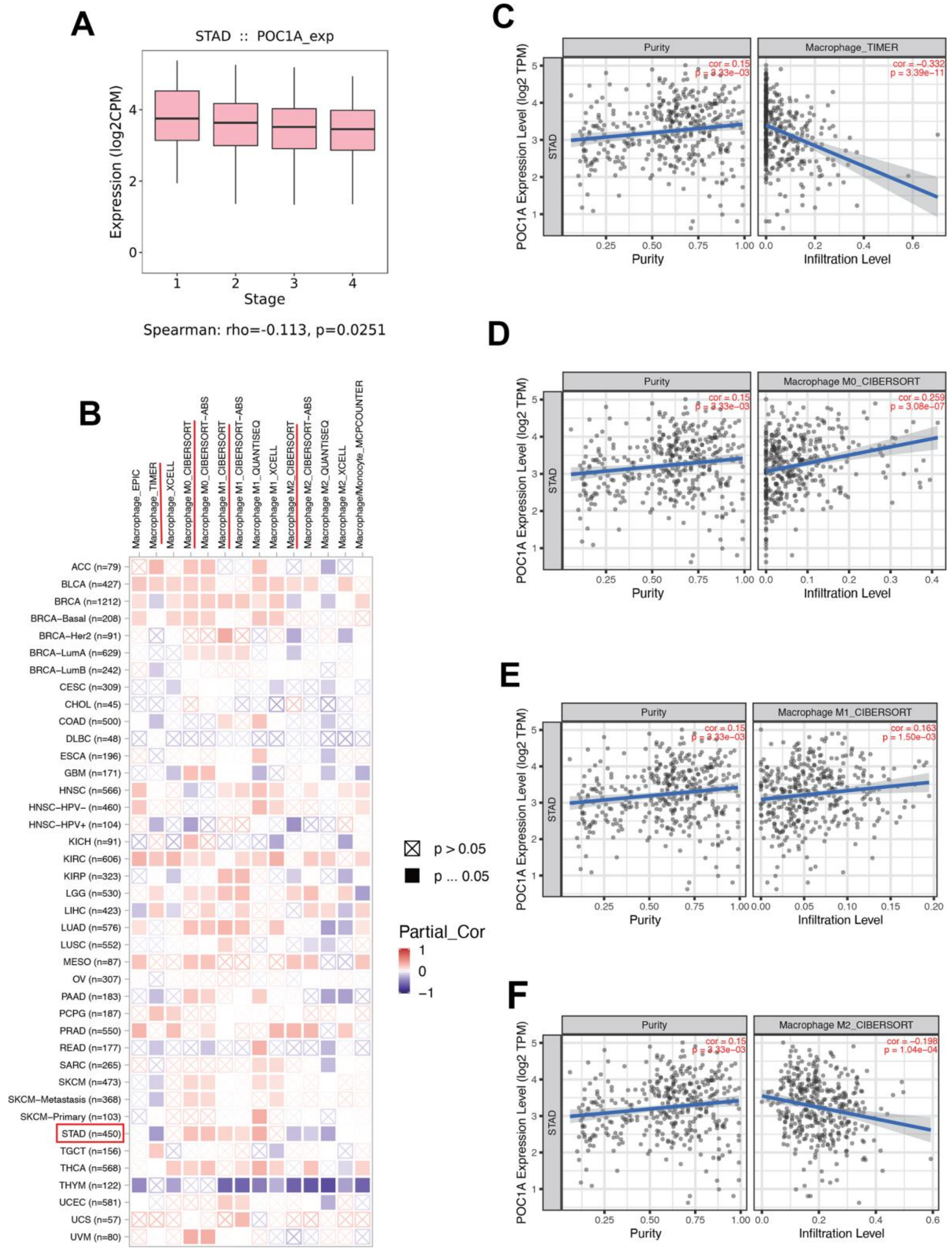

Supplementary Figure 5. The sense POC1A in clinical prognosis and immune infiltration. (**A**) Expression of POC1A had significant negative correlation with TNM stages (R=-0.1, p=0.02) according to the TISIDB; (**B**) Heatmap of correlation between POC1A expression and immune infiltration level of macrophages among different datasets and different cancer types; (**C–F**) Scatter plots of correlation between POC1A expression and immune infiltration of different macrophages in STAD.
