# Supplementary material for: POC1A acts as a promising prognostic biomarker associated with high tumor immune cell infiltration in gastric cancer
Source: Aging (Albany NY). 2020 Oct 14;12(19):18982–9011. doi: 10.18632/aging.103624 (PMC7732308; doi:10.18632/aging.103624)
Supplement: Supplementary Tables 1, 2, 3, 4 and 5 [file aging-12-103624-s002..pdf]

SUPPLEMENTARY TABLES

Supplementary Table 1. Primer sequence of 6 genes for RT-qPCR.

	Sequence (5'->3')	
**Genes**	**Forward Primer**	**Reverse Primer**
VMP1	GGTAGCTGCAGCCAGTACAA	TGCAACAACAGCAAGGGTTG
LAMP5	CCCCTGATTTTGGGGCTCAT	TACTGGGATCTGTCCCGAGG
ARMC9	CCGGATGCTACCACTTCTCG	AAAATCTTTGCGGGAAGCAGC
CEBPB	TTTGTCCAAACCAACCGCAC	CCCCCAAAAGGCTTTGTAACC
POC1A	GTGACAGCCTCTGACGACAA	AGAAAGTTGGGCATGGTGTTG
PAOX	CTGCTTCGGGGCTATCAAGG	TCGTGGAGTAAAACGTGCGA

Supplementary Table 2. Associations between POC1A mRNA levels and clinicopathological characteristics.

		Number	POC1A		p
			Low	High	
**Gender**					0.205
	Female	25	17	8	
	Male	76	61	15	
**Age (Years)**					0.709
	<=65	67	51	16	
	>65	34	27	7	
**T stage**					0.085
	T1	11	6	5	
	T2	6	4	2	
	T3	34	25	9	
	T4	50	43	7	
**N stage**					**0.001**
	N0	27	21	6	
	N1	9	2	7	
	N2	24	19	5	
	N3	31	36	5	
**TNM stage**					0.142
	I	14	8	6	
	II	22	17	5	
	III	65	53	12	
**Tumor Location**					0.486
	Upper	37	27	10	
	Middle	14	13	1	
	Lower	30	22	8	
	Diffuse	20	16	4	
**Tumor Size (mm)**					**0.043**
	<50	13	6	7	
	>=50	28	23	5	
**Differentiation grade**					0.249
	G1	14	13	1	
	G2	58	42	16	
	G3	29	23	6	

Supplementary Table 3. Associations between POC1A expression and clinicopathological characteristics in immunohistochemistry.

		Number	POC1A		p
			Low	High	
**Gender**					0.646
	Female	18	16	2	
	Male	73	59	14	
**Age (Years)**					0.368
	<=65	31	24	7	
	>65	60	51	9	
**BMI (kg/m2)**					0.646
	<=25	73	59	14	
	>25	18	16	2	
**T stage**					0.087
	T1	1	0	1	
	T2	10	7	3	
	T3	78	33	4	
	T4	2	35	8	
**N stage**					**0.04**
	N0	13	10	3	
	N1	16	14	2	
	N2	28	19	9	
	N3	34	32	2	
**M stage**					0.545
	M0	87	72	15	
	M1	4	3	1	
**TNM stage**					0.363
	I	4	3	1	
	II	23	17	6	
	III	60	52	8	
	IV	4	3	1	
**Position**					0.731
	Upper	31	25	6	
	Middle	17	14	3	
	Lower	36	31	5	
	Diffuse	7	5	2	
**Tumor Size (mm)**					**0.05**
	<50	48	36	12	
	>=50	43	39	4	
**Differentiation grade**					0.777
	Well-moderate	37	31	6	
	Poor-undifferentiation	54	44	10	
**Lymphatic or nervous invasion**					**0.036**
	Negative	59	45	14	
	Positive	32	30	2	
**Borrmann**					0.893
	Borrmann I-II	16	13	3	
	Borrmann III-IV	75	62	13	

Supplementary Table 4. Correlation between POC1A mRNA expression and clinical pathological features in GSE84433.

		Number	POC1A		p
			Low	High	
**Gender**					0.996
	Female	115	76	39	
	Male	242	160	82	
**Age (Years)**					0.836
	<=65	241	158	83	
	>65	116	78	38	
**T stage**					**0.002**
	T1	11	5	6	
	T2	35	16	19	
	T3	67	39	28	
	T4	244	176	68	
**N stage**					**0.008**
	N0	71	35	36	
	N1	155	107	48	
	N2	99	69	30	
	N3	32	25	7	

Supplementary Table 5. KEGG pathways of co-expression genes of POC1A.

ID	Description	GeneRatio	BgRatio	pvalue	p.adjust	qvalue	Count
hsa04110	Cell cycle	30/253	124/8016	6.54E-19	1.63E-16	1.45E-16	30
hsa03030	DNA replication	14/253	36/8016	1.39E-12	1.73E-10	1.54E-10	14
hsa04114	Oocyte meiosis	20/253	128/8016	2.59E-09	2.15E-07	1.91E-07	20
hsa04914	Progesterone-mediated oocyte maturation	17/253	99/8016	9.75E-09	6.07E-07	5.39E-07	17
hsa04022	cGMP-PKG signaling pathway	18/253	167/8016	4.83E-06	0.00024056	0.00021356	18
hsa03460	Fanconi anemia pathway	9/253	54/8016	4.14E-05	0.00171614	0.00152353	9
hsa05166	Human T-cell leukemia virus 1 infection	18/253	219/8016	0.00018402	0.00654571	0.00581103	18
hsa04115	p53 signaling pathway	9/253	72/8016	0.00040351	0.01175142	0.01043245	9
hsa04540	Gap junction	10/253	88/8016	0.00042475	0.01175142	0.01043245	10
hsa04218	Cellular senescence	14/253	160/8016	0.00051219	0.01275354	0.01132211	14
hsa03430	Mismatch repair	5/253	23/8016	0.00063523	0.01437923	0.01276532	5
hsa04270	Vascular smooth muscle contraction	12/253	132/8016	0.00091174	0.01891871	0.0167953	12
hsa04970	Salivary secretion	9/253	90/8016	0.00204635	0.03919542	0.03479619	9
hsa04730	Long-term depression	7/253	60/8016	0.00265927	0.04729696	0.04198842	7
