# Supplementary material for: POC1A acts as a promising prognostic biomarker associated with high tumor immune cell infiltration in gastric cancer
Source: Aging (Albany NY). 2020 Oct 14;12(19):18982–9011. doi: 10.18632/aging.103624 (PMC7732308; doi:10.18632/aging.103624)
Supplement: Supplementary Table 6 [file aging-12-103624-s003..docx]

**Supplementary Table 6** GO annotations of co-expression genes of POC1A

| **ID** | **Description** | **GeneRatio** | | **BgRatio** | **pvalue** | **p.adjust** | **qvalue** | **Count** | |
| --- | --- | --- | --- | --- | --- | --- | --- | --- | --- |
| GO:0140014 | mitotic nuclear division | | 60/561 | 264/18670 | 1.31E-35 | 5.42E-32 | 4.68E-32 | 60 |  |
| GO:0048285 | organelle fission | | 74/561 | 449/18670 | 8.10E-34 | 1.68E-30 | 1.45E-30 | 74 |  |
| GO:0007059 | chromosome segregation | | 62/561 | 321/18670 | 1.94E-32 | 2.55E-29 | 2.20E-29 | 62 |  |
| GO:0000280 | nuclear division | | 69/561 | 407/18670 | 2.46E-32 | 2.55E-29 | 2.20E-29 | 69 |  |
| GO:0000070 | mitotic sister chromatid segregation | | 43/561 | 151/18670 | 3.87E-30 | 3.20E-27 | 2.76E-27 | 43 |  |
| GO:0006260 | DNA replication | | 55/561 | 274/18670 | 9.66E-30 | 6.66E-27 | 5.75E-27 | 55 |  |
| GO:0006261 | DNA-dependent DNA replication | | 42/561 | 153/18670 | 9.56E-29 | 5.66E-26 | 4.88E-26 | 42 |  |
| GO:0000819 | sister chromatid segregation | | 45/561 | 189/18670 | 7.93E-28 | 4.10E-25 | 3.54E-25 | 45 |  |
| GO:0098813 | nuclear chromosome segregation | | 49/561 | 262/18670 | 3.94E-25 | 1.81E-22 | 1.56E-22 | 49 |  |
| GO:1902850 | microtubule cytoskeleton organization involved in mitosis | | 32/561 | 131/18670 | 1.77E-20 | 7.31E-18 | 6.30E-18 | 32 |  |
| GO:0007088 | regulation of mitotic nuclear division | | 33/561 | 164/18670 | 2.66E-18 | 1.00E-15 | 8.63E-16 | 33 |  |
| GO:0044786 | cell cycle DNA replication | | 23/561 | 71/18670 | 4.32E-18 | 1.49E-15 | 1.29E-15 | 23 |  |
| GO:0007052 | mitotic spindle organization | | 27/561 | 106/18670 | 5.53E-18 | 1.76E-15 | 1.52E-15 | 27 |  |
| GO:0007051 | spindle organization | | 33/561 | 170/18670 | 8.39E-18 | 2.48E-15 | 2.14E-15 | 33 |  |
| GO:0051983 | regulation of chromosome segregation | | 26/561 | 103/18670 | 2.93E-17 | 8.07E-15 | 6.96E-15 | 26 |  |
| GO:0071103 | DNA conformation change | | 44/561 | 327/18670 | 6.22E-17 | 1.61E-14 | 1.39E-14 | 44 |  |
| GO:0051783 | regulation of nuclear division | | 33/561 | 188/18670 | 1.97E-16 | 4.80E-14 | 4.14E-14 | 33 |  |
| GO:0033260 | nuclear DNA replication | | 20/561 | 60/18670 | 3.53E-16 | 8.12E-14 | 7.00E-14 | 20 |  |
| GO:0006270 | DNA replication initiation | | 16/561 | 37/18670 | 2.57E-15 | 5.60E-13 | 4.83E-13 | 16 |  |
| GO:0000075 | cell cycle checkpoint | | 33/561 | 216/18670 | 1.32E-14 | 2.74E-12 | 2.36E-12 | 33 |  |
| GO:0051304 | chromosome separation | | 22/561 | 90/18670 | 1.72E-14 | 3.39E-12 | 2.92E-12 | 22 |  |
| GO:0010965 | regulation of mitotic sister chromatid separation | | 18/561 | 57/18670 | 3.18E-14 | 5.99E-12 | 5.17E-12 | 18 |  |
| GO:0051306 | mitotic sister chromatid separation | | 18/561 | 60/18670 | 8.73E-14 | 1.57E-11 | 1.36E-11 | 18 |  |
| GO:0007093 | mitotic cell cycle checkpoint | | 28/561 | 165/18670 | 9.40E-14 | 1.62E-11 | 1.40E-11 | 28 |  |
| GO:1905818 | regulation of chromosome separation | | 18/561 | 62/18670 | 1.65E-13 | 2.69E-11 | 2.32E-11 | 18 |  |
| GO:0033045 | regulation of sister chromatid segregation | | 20/561 | 80/18670 | 1.70E-13 | 2.69E-11 | 2.32E-11 | 20 |  |
| GO:0007091 | metaphase/anaphase transition of mitotic cell cycle | | 17/561 | 54/18670 | 1.76E-13 | 2.69E-11 | 2.32E-11 | 17 |  |
| GO:1901990 | regulation of mitotic cell cycle phase transition | | 46/561 | 444/18670 | 2.46E-13 | 3.61E-11 | 3.11E-11 | 46 |  |
| GO:0044843 | cell cycle G1/S phase transition | | 37/561 | 298/18670 | 2.53E-13 | 3.61E-11 | 3.11E-11 | 37 |  |
| GO:1901987 | regulation of cell cycle phase transition | | 48/561 | 480/18670 | 2.73E-13 | 3.77E-11 | 3.25E-11 | 48 |  |
| GO:0044784 | metaphase/anaphase transition of cell cycle | | 17/561 | 56/18670 | 3.45E-13 | 4.61E-11 | 3.98E-11 | 17 |  |
| GO:0000082 | G1/S transition of mitotic cell cycle | | 35/561 | 279/18670 | 8.39E-13 | 1.08E-10 | 9.36E-11 | 35 |  |
| GO:0033047 | regulation of mitotic sister chromatid segregation | | 18/561 | 68/18670 | 9.61E-13 | 1.16E-10 | 9.97E-11 | 18 |  |
| GO:0030071 | regulation of mitotic metaphase/anaphase transition | | 16/561 | 51/18670 | 9.69E-13 | 1.16E-10 | 9.97E-11 | 16 |  |
| GO:0000727 | double-strand break repair via break-induced replication | | 9/561 | 11/18670 | 9.78E-13 | 1.16E-10 | 9.97E-11 | 9 |  |
| GO:0090068 | positive regulation of cell cycle process | | 36/561 | 298/18670 | 1.22E-12 | 1.41E-10 | 1.21E-10 | 36 |  |
| GO:0033046 | negative regulation of sister chromatid segregation | | 15/561 | 44/18670 | 1.26E-12 | 1.41E-10 | 1.21E-10 | 15 |  |
| GO:0045839 | negative regulation of mitotic nuclear division | | 16/561 | 52/18670 | 1.36E-12 | 1.48E-10 | 1.28E-10 | 16 |  |
| GO:0051985 | negative regulation of chromosome segregation | | 15/561 | 45/18670 | 1.83E-12 | 1.95E-10 | 1.68E-10 | 15 |  |
| GO:1902099 | regulation of metaphase/anaphase transition of cell cycle | | 16/561 | 53/18670 | 1.90E-12 | 1.96E-10 | 1.69E-10 | 16 |  |
| GO:0006271 | DNA strand elongation involved in DNA replication | | 11/561 | 20/18670 | 2.15E-12 | 2.17E-10 | 1.87E-10 | 11 |  |
| GO:0045930 | negative regulation of mitotic cell cycle | | 38/561 | 338/18670 | 2.68E-12 | 2.64E-10 | 2.28E-10 | 38 |  |
| GO:2000816 | negative regulation of mitotic sister chromatid separation | | 14/561 | 39/18670 | 3.15E-12 | 3.03E-10 | 2.61E-10 | 14 |  |
| GO:1905819 | negative regulation of chromosome separation | | 14/561 | 40/18670 | 4.71E-12 | 4.40E-10 | 3.80E-10 | 14 |  |
| GO:0010948 | negative regulation of cell cycle process | | 39/561 | 361/18670 | 4.79E-12 | 4.40E-10 | 3.80E-10 | 39 |  |
| GO:0022616 | DNA strand elongation | | 12/561 | 27/18670 | 5.56E-12 | 5.00E-10 | 4.31E-10 | 12 |  |
| GO:0033048 | negative regulation of mitotic sister chromatid segregation | | 14/561 | 42/18670 | 1.02E-11 | 8.94E-10 | 7.71E-10 | 14 |  |
| GO:0070507 | regulation of microtubule cytoskeleton organization | | 27/561 | 186/18670 | 1.19E-11 | 1.02E-09 | 8.76E-10 | 27 |  |
| GO:0045787 | positive regulation of cell cycle | | 40/561 | 389/18670 | 1.20E-11 | 1.02E-09 | 8.76E-10 | 40 |  |
| GO:0051784 | negative regulation of nuclear division | | 16/561 | 60/18670 | 1.57E-11 | 1.30E-09 | 1.12E-09 | 16 |  |
| GO:0045841 | negative regulation of mitotic metaphase/anaphase transition | | 13/561 | 36/18670 | 1.74E-11 | 1.41E-09 | 1.22E-09 | 13 |  |
| GO:0044839 | cell cycle G2/M phase transition | | 32/561 | 266/18670 | 2.51E-11 | 2.00E-09 | 1.72E-09 | 32 |  |
| GO:1902100 | negative regulation of metaphase/anaphase transition of cell cycle | | 13/561 | 37/18670 | 2.61E-11 | 2.04E-09 | 1.76E-09 | 13 |  |
| GO:1901988 | negative regulation of cell cycle phase transition | | 32/561 | 267/18670 | 2.77E-11 | 2.12E-09 | 1.83E-09 | 32 |  |
| GO:0032392 | DNA geometric change | | 21/561 | 119/18670 | 5.57E-11 | 4.19E-09 | 3.62E-09 | 21 |  |
| GO:1902969 | mitotic DNA replication | | 9/561 | 15/18670 | 7.99E-11 | 5.90E-09 | 5.09E-09 | 9 |  |
| GO:0000086 | G2/M transition of mitotic cell cycle | | 30/561 | 247/18670 | 8.36E-11 | 6.02E-09 | 5.20E-09 | 30 |  |
| GO:0000724 | double-strand break repair via homologous recombination | | 22/561 | 134/18670 | 8.44E-11 | 6.02E-09 | 5.20E-09 | 22 |  |
| GO:1903046 | meiotic cell cycle process | | 26/561 | 188/18670 | 8.67E-11 | 6.08E-09 | 5.24E-09 | 26 |  |
| GO:1901991 | negative regulation of mitotic cell cycle phase transition | | 30/561 | 248/18670 | 9.25E-11 | 6.38E-09 | 5.50E-09 | 30 |  |
| GO:0032886 | regulation of microtubule-based process | | 28/561 | 218/18670 | 9.62E-11 | 6.53E-09 | 5.63E-09 | 28 |  |
| GO:0000725 | recombinational repair | | 22/561 | 135/18670 | 9.81E-11 | 6.55E-09 | 5.65E-09 | 22 |  |
| GO:0051321 | meiotic cell cycle | | 30/561 | 249/18670 | 1.02E-10 | 6.72E-09 | 5.79E-09 | 30 |  |
| GO:0007094 | mitotic spindle assembly checkpoint | | 12/561 | 34/18670 | 1.45E-10 | 8.94E-09 | 7.71E-09 | 12 |  |
| GO:0031577 | spindle checkpoint | | 12/561 | 34/18670 | 1.45E-10 | 8.94E-09 | 7.71E-09 | 12 |  |
| GO:0071173 | spindle assembly checkpoint | | 12/561 | 34/18670 | 1.45E-10 | 8.94E-09 | 7.71E-09 | 12 |  |
| GO:0071174 | mitotic spindle checkpoint | | 12/561 | 34/18670 | 1.45E-10 | 8.94E-09 | 7.71E-09 | 12 |  |
| GO:0006302 | double-strand break repair | | 29/561 | 248/18670 | 4.30E-10 | 2.62E-08 | 2.26E-08 | 29 |  |
| GO:0032508 | DNA duplex unwinding | | 19/561 | 110/18670 | 6.74E-10 | 4.04E-08 | 3.49E-08 | 19 |  |
| GO:0045931 | positive regulation of mitotic cell cycle | | 23/561 | 163/18670 | 7.13E-10 | 4.22E-08 | 3.64E-08 | 23 |  |
| GO:0033044 | regulation of chromosome organization | | 34/561 | 342/18670 | 1.06E-09 | 6.18E-08 | 5.33E-08 | 34 |  |
| GO:0008608 | attachment of spindle microtubules to kinetochore | | 11/561 | 32/18670 | 1.19E-09 | 6.77E-08 | 5.84E-08 | 11 |  |
| GO:0006310 | DNA recombination | | 31/561 | 292/18670 | 1.19E-09 | 6.77E-08 | 5.84E-08 | 31 |  |
| GO:0031570 | DNA integrity checkpoint | | 22/561 | 157/18670 | 1.91E-09 | 1.07E-07 | 9.24E-08 | 22 |  |
| GO:0140013 | meiotic nuclear division | | 23/561 | 172/18670 | 2.09E-09 | 1.15E-07 | 9.93E-08 | 23 |  |
| GO:0051225 | spindle assembly | | 18/561 | 108/18670 | 3.44E-09 | 1.88E-07 | 1.62E-07 | 18 |  |
| GO:0006323 | DNA packaging | | 25/561 | 210/18670 | 4.82E-09 | 2.59E-07 | 2.23E-07 | 25 |  |
| GO:0090329 | regulation of DNA-dependent DNA replication | | 13/561 | 54/18670 | 5.08E-09 | 2.70E-07 | 2.33E-07 | 13 |  |
| GO:1902749 | regulation of cell cycle G2/M phase transition | | 25/561 | 213/18670 | 6.45E-09 | 3.38E-07 | 2.92E-07 | 25 |  |
| GO:0000083 | regulation of transcription involved in G1/S transition of mitotic cell cycle | | 10/561 | 29/18670 | 6.63E-09 | 3.43E-07 | 2.96E-07 | 10 |  |
| GO:0090307 | mitotic spindle assembly | | 13/561 | 56/18670 | 8.20E-09 | 4.19E-07 | 3.61E-07 | 13 |  |
| GO:0006275 | regulation of DNA replication | | 17/561 | 108/18670 | 2.28E-08 | 1.15E-06 | 9.93E-07 | 17 |  |
| GO:0010389 | regulation of G2/M transition of mitotic cell cycle | | 23/561 | 196/18670 | 2.62E-08 | 1.30E-06 | 1.13E-06 | 23 |  |
| GO:0065004 | protein-DNA complex assembly | | 26/561 | 248/18670 | 3.41E-08 | 1.68E-06 | 1.45E-06 | 26 |  |
| GO:0010639 | negative regulation of organelle organization | | 34/561 | 395/18670 | 4.06E-08 | 1.98E-06 | 1.71E-06 | 34 |  |
| GO:0036297 | interstrand cross-link repair | | 12/561 | 53/18670 | 4.18E-08 | 2.01E-06 | 1.73E-06 | 12 |  |
| GO:0007080 | mitotic metaphase plate congression | | 11/561 | 44/18670 | 5.10E-08 | 2.43E-06 | 2.09E-06 | 11 |  |
| GO:0006268 | DNA unwinding involved in DNA replication | | 7/561 | 14/18670 | 6.09E-08 | 2.86E-06 | 2.47E-06 | 7 |  |
| GO:0000079 | regulation of cyclin-dependent protein serine/threonine kinase activity | | 16/561 | 102/18670 | 6.21E-08 | 2.89E-06 | 2.49E-06 | 16 |  |
| GO:1901992 | positive regulation of mitotic cell cycle phase transition | | 15/561 | 91/18670 | 8.14E-08 | 3.75E-06 | 3.23E-06 | 15 |  |
| GO:0051052 | regulation of DNA metabolic process | | 35/561 | 429/18670 | 9.66E-08 | 4.39E-06 | 3.79E-06 | 35 |  |
| GO:0051310 | metaphase plate congression | | 12/561 | 57/18670 | 9.93E-08 | 4.47E-06 | 3.85E-06 | 12 |  |
| GO:1904029 | regulation of cyclin-dependent protein kinase activity | | 16/561 | 106/18670 | 1.08E-07 | 4.81E-06 | 4.15E-06 | 16 |  |
| GO:0071824 | protein-DNA complex subunit organization | | 27/561 | 288/18670 | 1.88E-07 | 8.30E-06 | 7.16E-06 | 27 |  |
| GO:0000076 | DNA replication checkpoint | | 7/561 | 16/18670 | 1.93E-07 | 8.39E-06 | 7.24E-06 | 7 |  |
| GO:0031109 | microtubule polymerization or depolymerization | | 16/561 | 112/18670 | 2.36E-07 | 1.01E-05 | 8.70E-06 | 16 |  |
| GO:0035637 | multicellular organismal signaling | | 22/561 | 204/18670 | 2.36E-07 | 1.01E-05 | 8.70E-06 | 22 |  |
| GO:0051303 | establishment of chromosome localization | | 13/561 | 75/18670 | 3.26E-07 | 1.38E-05 | 1.19E-05 | 13 |  |
| GO:0050000 | chromosome localization | | 13/561 | 76/18670 | 3.82E-07 | 1.60E-05 | 1.38E-05 | 13 |  |
| GO:0061337 | cardiac conduction | | 18/561 | 146/18670 | 4.09E-07 | 1.67E-05 | 1.44E-05 | 18 |  |
| GO:2001251 | negative regulation of chromosome organization | | 18/561 | 146/18670 | 4.09E-07 | 1.67E-05 | 1.44E-05 | 18 |  |
| GO:0045132 | meiotic chromosome segregation | | 14/561 | 90/18670 | 4.61E-07 | 1.87E-05 | 1.61E-05 | 14 |  |
| GO:0006336 | DNA replication-independent nucleosome assembly | | 11/561 | 54/18670 | 4.85E-07 | 1.95E-05 | 1.68E-05 | 11 |  |
| GO:0034080 | CENP-A containing nucleosome assembly | | 10/561 | 44/18670 | 5.47E-07 | 2.15E-05 | 1.85E-05 | 10 |  |
| GO:0061641 | CENP-A containing chromatin organization | | 10/561 | 44/18670 | 5.47E-07 | 2.15E-05 | 1.85E-05 | 10 |  |
| GO:0061982 | meiosis I cell cycle process | | 16/561 | 119/18670 | 5.50E-07 | 2.15E-05 | 1.85E-05 | 16 |  |
| GO:0034724 | DNA replication-independent nucleosome organization | | 11/561 | 55/18670 | 5.90E-07 | 2.28E-05 | 1.97E-05 | 11 |  |
| GO:0044774 | mitotic DNA integrity checkpoint | | 15/561 | 106/18670 | 6.36E-07 | 2.42E-05 | 2.08E-05 | 15 |  |
| GO:1901989 | positive regulation of cell cycle phase transition | | 15/561 | 106/18670 | 6.36E-07 | 2.42E-05 | 2.08E-05 | 15 |  |
| GO:0031145 | anaphase-promoting complex-dependent catabolic process | | 13/561 | 81/18670 | 8.19E-07 | 3.08E-05 | 2.66E-05 | 13 |  |
| GO:0034502 | protein localization to chromosome | | 13/561 | 82/18670 | 9.47E-07 | 3.53E-05 | 3.05E-05 | 13 |  |
| GO:0007098 | centrosome cycle | | 16/561 | 124/18670 | 9.68E-07 | 3.58E-05 | 3.08E-05 | 16 |  |
| GO:0000910 | cytokinesis | | 19/561 | 171/18670 | 9.91E-07 | 3.63E-05 | 3.13E-05 | 19 |  |
| GO:0031055 | chromatin remodeling at centromere | | 10/561 | 48/18670 | 1.30E-06 | 4.70E-05 | 4.06E-05 | 10 |  |
| GO:0000281 | mitotic cytokinesis | | 12/561 | 73/18670 | 1.67E-06 | 5.90E-05 | 5.09E-05 | 12 |  |
| GO:0072401 | signal transduction involved in DNA integrity checkpoint | | 12/561 | 73/18670 | 1.67E-06 | 5.90E-05 | 5.09E-05 | 12 |  |
| GO:0072422 | signal transduction involved in DNA damage checkpoint | | 12/561 | 73/18670 | 1.67E-06 | 5.90E-05 | 5.09E-05 | 12 |  |
| GO:0071459 | protein localization to chromosome, centromeric region | | 7/561 | 21/18670 | 1.72E-06 | 6.02E-05 | 5.20E-05 | 7 |  |
| GO:0000723 | telomere maintenance | | 18/561 | 162/18670 | 1.90E-06 | 6.63E-05 | 5.71E-05 | 18 |  |
| GO:0072395 | signal transduction involved in cell cycle checkpoint | | 12/561 | 74/18670 | 1.94E-06 | 6.68E-05 | 5.76E-05 | 12 |  |
| GO:0007062 | sister chromatid cohesion | | 11/561 | 63/18670 | 2.44E-06 | 8.36E-05 | 7.21E-05 | 11 |  |
| GO:0031497 | chromatin assembly | | 18/561 | 165/18670 | 2.48E-06 | 8.36E-05 | 7.21E-05 | 18 |  |
| GO:0031023 | microtubule organizing center organization | | 16/561 | 133/18670 | 2.49E-06 | 8.36E-05 | 7.21E-05 | 16 |  |
| GO:0051302 | regulation of cell division | | 18/561 | 168/18670 | 3.22E-06 | 0.00010741 | 9.26E-05 | 18 |  |
| GO:0086012 | membrane depolarization during cardiac muscle cell action potential | | 7/561 | 23/18670 | 3.44E-06 | 0.00011379 | 9.81E-05 | 7 |  |
| GO:0006936 | muscle contraction | | 28/561 | 360/18670 | 4.70E-06 | 0.00015446 | 0.00013322 | 28 |  |
| GO:0007019 | microtubule depolymerization | | 9/561 | 44/18670 | 5.19E-06 | 0.00016926 | 0.00014597 | 9 |  |
| GO:0051231 | spindle elongation | | 5/561 | 10/18670 | 5.35E-06 | 0.00017308 | 0.00014927 | 5 |  |
| GO:0034508 | centromere complex assembly | | 10/561 | 56/18670 | 5.68E-06 | 0.00018237 | 0.00015729 | 10 |  |
| GO:0032200 | telomere organization | | 18/561 | 175/18670 | 5.74E-06 | 0.00018291 | 0.00015775 | 18 |  |
| GO:0034501 | protein localization to kinetochore | | 6/561 | 17/18670 | 6.69E-06 | 0.0002097 | 0.00018085 | 6 |  |
| GO:0046068 | cGMP metabolic process | | 6/561 | 17/18670 | 6.69E-06 | 0.0002097 | 0.00018085 | 6 |  |
| GO:0000077 | DNA damage checkpoint | | 16/561 | 145/18670 | 7.70E-06 | 0.00023796 | 0.00020523 | 16 |  |
| GO:0006334 | nucleosome assembly | | 16/561 | 145/18670 | 7.70E-06 | 0.00023796 | 0.00020523 | 16 |  |
| GO:0007127 | meiosis I | | 14/561 | 114/18670 | 8.24E-06 | 0.00025275 | 0.00021798 | 14 |  |
| GO:0061640 | cytoskeleton-dependent cytokinesis | | 13/561 | 100/18670 | 9.22E-06 | 0.00028063 | 0.00024203 | 13 |  |
| GO:1903779 | regulation of cardiac conduction | | 11/561 | 73/18670 | 1.08E-05 | 0.00032409 | 0.00027951 | 11 |  |
| GO:0043486 | histone exchange | | 10/561 | 60/18670 | 1.08E-05 | 0.00032409 | 0.00027951 | 10 |  |
| GO:0010971 | positive regulation of G2/M transition of mitotic cell cycle | | 7/561 | 27/18670 | 1.12E-05 | 0.00033149 | 0.00028589 | 7 |  |
| GO:0032201 | telomere maintenance via semi-conservative replication | | 7/561 | 27/18670 | 1.12E-05 | 0.00033149 | 0.00028589 | 7 |  |
| GO:0086010 | membrane depolarization during action potential | | 8/561 | 37/18670 | 1.13E-05 | 0.00033201 | 0.00028634 | 8 |  |
| GO:0003012 | muscle system process | | 32/561 | 465/18670 | 1.28E-05 | 0.00037249 | 0.00032125 | 32 |  |
| GO:0032465 | regulation of cytokinesis | | 12/561 | 89/18670 | 1.39E-05 | 0.00040104 | 0.00034587 | 12 |  |
| GO:0016572 | histone phosphorylation | | 8/561 | 38/18670 | 1.39E-05 | 0.00040104 | 0.00034587 | 8 |  |
| GO:1902750 | negative regulation of cell cycle G2/M phase transition | | 13/561 | 105/18670 | 1.58E-05 | 0.00045052 | 0.00038855 | 13 |  |
| GO:0007077 | mitotic nuclear envelope disassembly | | 5/561 | 12/18670 | 1.60E-05 | 0.00045052 | 0.00038855 | 5 |  |
| GO:0051988 | regulation of attachment of spindle microtubules to kinetochore | | 5/561 | 12/18670 | 1.60E-05 | 0.00045052 | 0.00038855 | 5 |  |
| GO:0006333 | chromatin assembly or disassembly | | 18/561 | 191/18670 | 1.92E-05 | 0.00053841 | 0.00046435 | 18 |  |
| GO:0072331 | signal transduction by p53 class mediator | | 22/561 | 267/18670 | 2.02E-05 | 0.00056077 | 0.00048364 | 22 |  |
| GO:0009187 | cyclic nucleotide metabolic process | | 8/561 | 40/18670 | 2.08E-05 | 0.00057426 | 0.00049527 | 8 |  |
| GO:1902751 | positive regulation of cell cycle G2/M phase transition | | 7/561 | 30/18670 | 2.38E-05 | 0.00065152 | 0.0005619 | 7 |  |
| GO:0045840 | positive regulation of mitotic nuclear division | | 9/561 | 53/18670 | 2.55E-05 | 0.0006951 | 0.00059949 | 9 |  |
| GO:0051383 | kinetochore organization | | 6/561 | 21/18670 | 2.65E-05 | 0.00071589 | 0.00061741 | 6 |  |
| GO:0071897 | DNA biosynthetic process | | 18/561 | 196/18670 | 2.73E-05 | 0.00073305 | 0.00063222 | 18 |  |
| GO:0044773 | mitotic DNA damage checkpoint | | 12/561 | 97/18670 | 3.36E-05 | 0.00089724 | 0.00077382 | 12 |  |
| GO:1904666 | regulation of ubiquitin protein ligase activity | | 6/561 | 22/18670 | 3.55E-05 | 0.000941 | 0.00081156 | 6 |  |
| GO:0007100 | mitotic centrosome separation | | 5/561 | 14/18670 | 3.85E-05 | 0.00099525 | 0.00085835 | 5 |  |
| GO:0090231 | regulation of spindle checkpoint | | 5/561 | 14/18670 | 3.85E-05 | 0.00099525 | 0.00085835 | 5 |  |
| GO:0090266 | regulation of mitotic cell cycle spindle assembly checkpoint | | 5/561 | 14/18670 | 3.85E-05 | 0.00099525 | 0.00085835 | 5 |  |
| GO:1903504 | regulation of mitotic spindle checkpoint | | 5/561 | 14/18670 | 3.85E-05 | 0.00099525 | 0.00085835 | 5 |  |
| GO:0006977 | DNA damage response, signal transduction by p53 class mediator resulting in cell cycle arrest | | 9/561 | 56/18670 | 4.03E-05 | 0.00103561 | 0.00089315 | 9 |  |
| GO:0034728 | nucleosome organization | | 17/561 | 184/18670 | 4.20E-05 | 0.00107255 | 0.00092502 | 17 |  |
| GO:0040001 | establishment of mitotic spindle localization | | 7/561 | 33/18670 | 4.61E-05 | 0.00116841 | 0.00100769 | 7 |  |
| GO:0072431 | signal transduction involved in mitotic G1 DNA damage checkpoint | | 9/561 | 57/18670 | 4.66E-05 | 0.00116841 | 0.00100769 | 9 |  |
| GO:1902400 | intracellular signal transduction involved in G1 DNA damage checkpoint | | 9/561 | 57/18670 | 4.66E-05 | 0.00116841 | 0.00100769 | 9 |  |
| GO:0051299 | centrosome separation | | 5/561 | 15/18670 | 5.63E-05 | 0.00140342 | 0.00121037 | 5 |  |
| GO:0072413 | signal transduction involved in mitotic cell cycle checkpoint | | 9/561 | 59/18670 | 6.17E-05 | 0.00151066 | 0.00130286 | 9 |  |
| GO:1902402 | signal transduction involved in mitotic DNA damage checkpoint | | 9/561 | 59/18670 | 6.17E-05 | 0.00151066 | 0.00130286 | 9 |  |
| GO:1902403 | signal transduction involved in mitotic DNA integrity checkpoint | | 9/561 | 59/18670 | 6.17E-05 | 0.00151066 | 0.00130286 | 9 |  |
| GO:0051054 | positive regulation of DNA metabolic process | | 19/561 | 228/18670 | 6.28E-05 | 0.00152967 | 0.00131925 | 19 |  |
| GO:0008016 | regulation of heart contraction | | 20/561 | 251/18670 | 7.60E-05 | 0.00183942 | 0.0015864 | 20 |  |
| GO:0030397 | membrane disassembly | | 5/561 | 16/18670 | 7.98E-05 | 0.00189947 | 0.00163819 | 5 |  |
| GO:0051081 | nuclear envelope disassembly | | 5/561 | 16/18670 | 7.98E-05 | 0.00189947 | 0.00163819 | 5 |  |
| GO:0055012 | ventricular cardiac muscle cell differentiation | | 5/561 | 16/18670 | 7.98E-05 | 0.00189947 | 0.00163819 | 5 |  |
| GO:0055023 | positive regulation of cardiac muscle tissue growth | | 8/561 | 48/18670 | 8.27E-05 | 0.00195556 | 0.00168657 | 8 |  |
| GO:0051899 | membrane depolarization | | 11/561 | 91/18670 | 8.79E-05 | 0.00206853 | 0.001784 | 11 |  |
| GO:0055025 | positive regulation of cardiac muscle tissue development | | 9/561 | 62/18670 | 9.19E-05 | 0.00214991 | 0.00185418 | 9 |  |
| GO:1903522 | regulation of blood circulation | | 22/561 | 297/18670 | 9.99E-05 | 0.00231634 | 0.00199772 | 22 |  |
| GO:0031111 | negative regulation of microtubule polymerization or depolymerization | | 7/561 | 37/18670 | 0.00010015 | 0.00231634 | 0.00199772 | 7 |  |
| GO:0031571 | mitotic G1 DNA damage checkpoint | | 9/561 | 63/18670 | 0.00010442 | 0.0023884 | 0.00205986 | 9 |  |
| GO:0044819 | mitotic G1/S transition checkpoint | | 9/561 | 63/18670 | 0.00010442 | 0.0023884 | 0.00205986 | 9 |  |
| GO:0007050 | cell cycle arrest | | 19/561 | 237/18670 | 0.00010526 | 0.00239428 | 0.00206494 | 19 |  |
| GO:0007076 | mitotic chromosome condensation | | 5/561 | 17/18670 | 0.00011031 | 0.00249556 | 0.00215228 | 5 |  |
| GO:0044783 | G1 DNA damage checkpoint | | 9/561 | 64/18670 | 0.00011832 | 0.00266216 | 0.00229597 | 9 |  |
| GO:0031110 | regulation of microtubule polymerization or depolymerization | | 10/561 | 79/18670 | 0.0001239 | 0.00277269 | 0.0023913 | 10 |  |
| GO:0042391 | regulation of membrane potential | | 28/561 | 434/18670 | 0.00013406 | 0.00298388 | 0.00257343 | 28 |  |
| GO:0033314 | mitotic DNA replication checkpoint | | 4/561 | 10/18670 | 0.00014661 | 0.00321154 | 0.00276978 | 4 |  |
| GO:0044557 | relaxation of smooth muscle | | 4/561 | 10/18670 | 0.00014661 | 0.00321154 | 0.00276978 | 4 |  |
| GO:0051255 | spindle midzone assembly | | 4/561 | 10/18670 | 0.00014661 | 0.00321154 | 0.00276978 | 4 |  |
| GO:0060421 | positive regulation of heart growth | | 8/561 | 52/18670 | 0.00014837 | 0.00323296 | 0.00278826 | 8 |  |
| GO:0051785 | positive regulation of nuclear division | | 9/561 | 66/18670 | 0.00015078 | 0.0032683 | 0.00281873 | 9 |  |
| GO:0051984 | positive regulation of chromosome segregation | | 6/561 | 28/18670 | 0.00015358 | 0.00331148 | 0.00285597 | 6 |  |
| GO:2000573 | positive regulation of DNA biosynthetic process | | 9/561 | 67/18670 | 0.00016961 | 0.00362297 | 0.00312461 | 9 |  |
| GO:0071158 | positive regulation of cell cycle arrest | | 10/561 | 82/18670 | 0.00016977 | 0.00362297 | 0.00312461 | 10 |  |
| GO:0050848 | regulation of calcium-mediated signaling | | 11/561 | 98/18670 | 0.00017237 | 0.00365953 | 0.00315615 | 11 |  |
| GO:0042770 | signal transduction in response to DNA damage | | 13/561 | 133/18670 | 0.00018806 | 0.00397225 | 0.00342585 | 13 |  |
| GO:0019985 | translesion synthesis | | 7/561 | 41/18670 | 0.00019712 | 0.00414243 | 0.00357262 | 7 |  |
| GO:0035404 | histone-serine phosphorylation | | 4/561 | 11/18670 | 0.00022493 | 0.00470299 | 0.00405608 | 4 |  |
| GO:0044818 | mitotic G2/M transition checkpoint | | 6/561 | 30/18670 | 0.00023001 | 0.00478514 | 0.00412693 | 6 |  |
| GO:0086065 | cell communication involved in cardiac conduction | | 8/561 | 56/18670 | 0.00025211 | 0.00521872 | 0.00450086 | 8 |  |
| GO:0051293 | establishment of spindle localization | | 7/561 | 43/18670 | 0.00026825 | 0.00552513 | 0.00476512 | 7 |  |
| GO:0007143 | female meiotic nuclear division | | 6/561 | 31/18670 | 0.00027804 | 0.00561497 | 0.00484261 | 6 |  |
| GO:0031572 | G2 DNA damage checkpoint | | 6/561 | 31/18670 | 0.00027804 | 0.00561497 | 0.00484261 | 6 |  |
| GO:0045737 | positive regulation of cyclin-dependent protein serine/threonine kinase activity | | 6/561 | 31/18670 | 0.00027804 | 0.00561497 | 0.00484261 | 6 |  |
| GO:0090075 | relaxation of muscle | | 6/561 | 31/18670 | 0.00027804 | 0.00561497 | 0.00484261 | 6 |  |
| GO:0043044 | ATP-dependent chromatin remodeling | | 10/561 | 88/18670 | 0.00030507 | 0.006131 | 0.00528765 | 10 |  |
| GO:0097553 | calcium ion transmembrane import into cytosol | | 13/561 | 140/18670 | 0.00031243 | 0.00624866 | 0.00538914 | 13 |  |
| GO:0046605 | regulation of centrosome cycle | | 8/561 | 58/18670 | 0.00032279 | 0.00642477 | 0.00554101 | 8 |  |
| GO:0060047 | heart contraction | | 20/561 | 280/18670 | 0.0003288 | 0.00646292 | 0.00557392 | 20 |  |
| GO:1904668 | positive regulation of ubiquitin protein ligase activity | | 4/561 | 12/18670 | 0.00032939 | 0.00646292 | 0.00557392 | 4 |  |
| GO:2000105 | positive regulation of DNA-dependent DNA replication | | 4/561 | 12/18670 | 0.00032939 | 0.00646292 | 0.00557392 | 4 |  |
| GO:0010880 | regulation of release of sequestered calcium ion into cytosol by sarcoplasmic reticulum | | 6/561 | 32/18670 | 0.00033359 | 0.00648391 | 0.00559202 | 6 |  |
| GO:1901976 | regulation of cell cycle checkpoint | | 6/561 | 32/18670 | 0.00033359 | 0.00648391 | 0.00559202 | 6 |  |
| GO:0010569 | regulation of double-strand break repair via homologous recombination | | 7/561 | 45/18670 | 0.00035868 | 0.00693898 | 0.0059845 | 7 |  |
| GO:2000134 | negative regulation of G1/S transition of mitotic cell cycle | | 12/561 | 125/18670 | 0.00038846 | 0.0074801 | 0.00645118 | 12 |  |
| GO:0071156 | regulation of cell cycle arrest | | 11/561 | 108/18670 | 0.00040545 | 0.00777111 | 0.00670216 | 11 |  |
| GO:1902806 | regulation of cell cycle G1/S phase transition | | 16/561 | 202/18670 | 0.00041367 | 0.00789211 | 0.00680652 | 16 |  |
| GO:2000045 | regulation of G1/S transition of mitotic cell cycle | | 15/561 | 184/18670 | 0.00045853 | 0.00870783 | 0.00751004 | 15 |  |
| GO:0051315 | attachment of mitotic spindle microtubules to kinetochore | | 4/561 | 13/18670 | 0.00046452 | 0.0087813 | 0.0075734 | 4 |  |
| GO:0030261 | chromosome condensation | | 7/561 | 47/18670 | 0.00047201 | 0.0088824 | 0.00766059 | 7 |  |
| GO:0006939 | smooth muscle contraction | | 11/561 | 110/18670 | 0.00047483 | 0.00889507 | 0.00767152 | 11 |  |
| GO:0010972 | negative regulation of G2/M transition of mitotic cell cycle | | 10/561 | 93/18670 | 0.00047778 | 0.00890995 | 0.00768435 | 10 |  |
| GO:2000779 | regulation of double-strand break repair | | 9/561 | 77/18670 | 0.00049085 | 0.00911259 | 0.00785912 | 9 |  |
| GO:0055001 | muscle cell development | | 15/561 | 186/18670 | 0.00051386 | 0.00948354 | 0.00817904 | 15 |  |
| GO:0003015 | heart process | | 20/561 | 290/18670 | 0.00051541 | 0.00948354 | 0.00817904 | 20 |  |
| GO:0051653 | spindle localization | | 7/561 | 48/18670 | 0.00053849 | 0.00985525 | 0.00849962 | 7 |  |
| GO:0055021 | regulation of cardiac muscle tissue growth | | 9/561 | 78/18670 | 0.00054037 | 0.00985525 | 0.00849962 | 9 |  |
| GO:0098687 | chromosomal region | | 61/589 | 349/19717 | 1.77E-29 | 8.52E-27 | 6.90E-27 | 61 |  |
| GO:0000775 | chromosome, centromeric region | | 43/589 | 193/19717 | 1.89E-25 | 4.54E-23 | 3.68E-23 | 43 |  |
| GO:0000779 | condensed chromosome, centromeric region | | 32/589 | 118/19717 | 4.71E-22 | 7.56E-20 | 6.12E-20 | 32 |  |
| GO:0005819 | spindle | | 51/589 | 347/19717 | 2.95E-21 | 3.54E-19 | 2.87E-19 | 51 |  |
| GO:0000776 | kinetochore | | 33/589 | 135/19717 | 3.75E-21 | 3.61E-19 | 2.92E-19 | 33 |  |
| GO:0000793 | condensed chromosome | | 40/589 | 223/19717 | 4.39E-20 | 3.52E-18 | 2.85E-18 | 40 |  |
| GO:0000777 | condensed chromosome kinetochore | | 28/589 | 105/19717 | 3.07E-19 | 2.11E-17 | 1.71E-17 | 28 |  |
| GO:0005874 | microtubule | | 47/589 | 416/19717 | 4.38E-15 | 2.64E-13 | 2.13E-13 | 47 |  |
| GO:0072686 | mitotic spindle | | 24/589 | 109/19717 | 1.24E-14 | 6.61E-13 | 5.35E-13 | 24 |  |
| GO:0000780 | condensed nuclear chromosome, centromeric region | | 11/589 | 26/19717 | 7.93E-11 | 3.81E-09 | 3.09E-09 | 11 |  |
| GO:0000794 | condensed nuclear chromosome | | 19/589 | 99/19717 | 9.32E-11 | 4.07E-09 | 3.30E-09 | 19 |  |
| GO:0000922 | spindle pole | | 24/589 | 164/19717 | 1.27E-10 | 5.09E-09 | 4.12E-09 | 24 |  |
| GO:0030496 | midbody | | 24/589 | 173/19717 | 3.92E-10 | 1.45E-08 | 1.17E-08 | 24 |  |
| GO:0005876 | spindle microtubule | | 14/589 | 59/19717 | 1.49E-09 | 5.12E-08 | 4.15E-08 | 14 |  |
| GO:0000778 | condensed nuclear chromosome kinetochore | | 8/589 | 15/19717 | 3.24E-09 | 1.04E-07 | 8.40E-08 | 8 |  |
| GO:0042555 | MCM complex | | 7/589 | 12/19717 | 1.42E-08 | 4.28E-07 | 3.47E-07 | 7 |  |
| GO:0005657 | replication fork | | 14/589 | 70/19717 | 1.60E-08 | 4.53E-07 | 3.67E-07 | 14 |  |
| GO:0005875 | microtubule associated complex | | 20/589 | 152/19717 | 2.85E-08 | 7.61E-07 | 6.16E-07 | 20 |  |
| GO:0000940 | condensed chromosome outer kinetochore | | 7/589 | 14/19717 | 5.86E-08 | 1.48E-06 | 1.20E-06 | 7 |  |
| GO:0051233 | spindle midzone | | 9/589 | 34/19717 | 4.79E-07 | 1.15E-05 | 9.34E-06 | 9 |  |
| GO:0005871 | kinesin complex | | 11/589 | 55/19717 | 5.59E-07 | 1.28E-05 | 1.04E-05 | 11 |  |
| GO:0000781 | chromosome, telomeric region | | 18/589 | 161/19717 | 1.62E-06 | 3.53E-05 | 2.86E-05 | 18 |  |
| GO:0043596 | nuclear replication fork | | 9/589 | 41/19717 | 2.66E-06 | 5.56E-05 | 4.50E-05 | 9 |  |
| GO:0000307 | cyclin-dependent protein kinase holoenzyme complex | | 9/589 | 42/19717 | 3.29E-06 | 6.60E-05 | 5.34E-05 | 9 |  |
| GO:1990752 | microtubule end | | 7/589 | 29/19717 | 1.80E-05 | 0.00034699 | 0.00028097 | 7 |  |
| GO:0010369 | chromocenter | | 5/589 | 14/19717 | 3.74E-05 | 0.00069246 | 0.0005607 | 5 |  |
| GO:0042383 | sarcolemma | | 14/589 | 136/19717 | 5.78E-05 | 0.00103011 | 0.0008341 | 14 |  |
| GO:0043292 | contractile fiber | | 19/589 | 234/19717 | 8.29E-05 | 0.00142449 | 0.00115343 | 19 |  |
| GO:0000784 | nuclear chromosome, telomeric region | | 13/589 | 125/19717 | 9.50E-05 | 0.00157511 | 0.0012754 | 13 |  |
| GO:0014704 | intercalated disc | | 8/589 | 50/19717 | 0.00010734 | 0.00172101 | 0.00139353 | 8 |  |
| GO:0044449 | contractile fiber part | | 18/589 | 221/19717 | 0.00012172 | 0.00188858 | 0.00152922 | 18 |  |
| GO:0005901 | caveola | | 10/589 | 80/19717 | 0.00013177 | 0.00198063 | 0.00160375 | 10 |  |
| GO:0030315 | T-tubule | | 8/589 | 52/19717 | 0.00014278 | 0.00204097 | 0.00165261 | 8 |  |
| GO:0030016 | myofibril | | 18/589 | 224/19717 | 0.00014427 | 0.00204097 | 0.00165261 | 18 |  |
| GO:0035371 | microtubule plus-end | | 5/589 | 20/19717 | 0.0002498 | 0.0034065 | 0.0027583 | 5 |  |
| GO:0044291 | cell-cell contact zone | | 9/589 | 71/19717 | 0.00025496 | 0.0034065 | 0.0027583 | 9 |  |
| GO:0030017 | sarcomere | | 16/589 | 204/19717 | 0.00043482 | 0.00560589 | 0.00453918 | 16 |  |
| GO:0043240 | Fanconi anaemia nuclear complex | | 4/589 | 13/19717 | 0.00045453 | 0.00560589 | 0.00453918 | 4 |  |
| GO:1990023 | mitotic spindle midzone | | 4/589 | 13/19717 | 0.00045453 | 0.00560589 | 0.00453918 | 4 |  |
| GO:0032155 | cell division site part | | 8/589 | 63/19717 | 0.00055133 | 0.00662969 | 0.00536817 | 8 |  |
| GO:0016528 | sarcoplasm | | 9/589 | 80/19717 | 0.00062624 | 0.0073469 | 0.00594891 | 9 |  |
| GO:0090533 | cation-transporting ATPase complex | | 4/589 | 15/19717 | 0.00082741 | 0.00947581 | 0.00767272 | 4 |  |
| GO:0032153 | cell division site | | 8/589 | 68/19717 | 0.00092385 | 0.01033418 | 0.00836776 | 8 |  |
| GO:0032154 | cleavage furrow | | 7/589 | 55/19717 | 0.00119897 | 0.01279614 | 0.01036125 | 7 |  |
| GO:0031674 | I band | | 12/589 | 143/19717 | 0.00122943 | 0.01279614 | 0.01036125 | 12 |  |
| GO:0016529 | sarcoplasmic reticulum | | 8/589 | 71/19717 | 0.00122992 | 0.01279614 | 0.01036125 | 8 |  |
| GO:1902554 | serine/threonine protein kinase complex | | 9/589 | 88/19717 | 0.00125035 | 0.01279614 | 0.01036125 | 9 |  |
| GO:0005911 | cell-cell junction | | 26/589 | 459/19717 | 0.00146717 | 0.01470229 | 0.01190469 | 26 |  |
| GO:0044853 | plasma membrane raft | | 10/589 | 109/19717 | 0.00157984 | 0.01550826 | 0.0125573 | 10 |  |
| GO:0032432 | actin filament bundle | | 8/589 | 75/19717 | 0.00175802 | 0.01691213 | 0.01369403 | 8 |  |
| GO:0045171 | intercellular bridge | | 7/589 | 59/19717 | 0.00181934 | 0.01715886 | 0.01389381 | 7 |  |
| GO:0030018 | Z disc | | 11/589 | 132/19717 | 0.00204688 | 0.01893364 | 0.01533088 | 11 |  |
| GO:0000790 | nuclear chromatin | | 22/589 | 377/19717 | 0.00229496 | 0.02082785 | 0.01686465 | 22 |  |
| GO:0034703 | cation channel complex | | 15/589 | 220/19717 | 0.00262437 | 0.02337635 | 0.01892822 | 15 |  |
| GO:0005814 | centriole | | 11/589 | 139/19717 | 0.00306759 | 0.02655671 | 0.02150341 | 11 |  |
| GO:0005680 | anaphase-promoting complex | | 4/589 | 21/19717 | 0.00314705 | 0.02655671 | 0.02150341 | 4 |  |
| GO:0098533 | ATPase dependent transmembrane transport complex | | 4/589 | 21/19717 | 0.00314705 | 0.02655671 | 0.02150341 | 4 |  |
| GO:0120111 | neuron projection cytoplasm | | 8/589 | 84/19717 | 0.00359873 | 0.02981319 | 0.02414023 | 8 |  |
| GO:0005652 | nuclear lamina | | 3/589 | 11/19717 | 0.00365797 | 0.02981319 | 0.02414023 | 3 |  |
| GO:0001725 | stress fiber | | 7/589 | 67/19717 | 0.00378088 | 0.02981319 | 0.02414023 | 7 |  |
| GO:0097517 | contractile actin filament bundle | | 7/589 | 67/19717 | 0.00378088 | 0.02981319 | 0.02414023 | 7 |  |
| GO:0043198 | dendritic shaft | | 5/589 | 36/19717 | 0.00409864 | 0.03179754 | 0.025747 | 5 |  |
| GO:1902495 | transmembrane transporter complex | | 19/589 | 324/19717 | 0.00419901 | 0.03205912 | 0.0259588 | 19 |  |
| GO:0030687 | preribosome, large subunit precursor | | 4/589 | 23/19717 | 0.00444155 | 0.03338103 | 0.02702917 | 4 |  |
| GO:0005890 | sodium:potassium-exchanging ATPase complex | | 3/589 | 12/19717 | 0.00476987 | 0.03529703 | 0.02858059 | 3 |  |
| GO:0043601 | nuclear replisome | | 4/589 | 24/19717 | 0.00520578 | 0.0379391 | 0.03071992 | 4 |  |
| GO:1902911 | protein kinase complex | | 9/589 | 109/19717 | 0.00538814 | 0.03845406 | 0.03113689 | 9 |  |
| GO:1990351 | transporter complex | | 19/589 | 332/19717 | 0.00543633 | 0.03845406 | 0.03113689 | 19 |  |
| GO:0031672 | A band | | 5/589 | 39/19717 | 0.0058178 | 0.040556 | 0.03283886 | 5 |  |
| GO:0034399 | nuclear periphery | | 10/589 | 131/19717 | 0.00598635 | 0.04113475 | 0.03330749 | 10 |  |
| GO:0030894 | replisome | | 4/589 | 26/19717 | 0.00698766 | 0.04733892 | 0.03833111 | 4 |  |
| GO:0008017 | microtubule binding | | 33/553 | 246/17697 | 1.64E-12 | 1.07E-09 | 9.80E-10 | 33 |  |
| GO:0140097 | catalytic activity, acting on DNA | | 30/553 | 213/17697 | 4.90E-12 | 1.51E-09 | 1.39E-09 | 30 |  |
| GO:0015631 | tubulin binding | | 38/553 | 336/17697 | 6.99E-12 | 1.51E-09 | 1.39E-09 | 38 |  |
| GO:0003688 | DNA replication origin binding | | 11/553 | 24/17697 | 4.34E-11 | 5.85E-09 | 5.37E-09 | 11 |  |
| GO:0003678 | DNA helicase activity | | 18/553 | 81/17697 | 4.51E-11 | 5.85E-09 | 5.37E-09 | 18 |  |
| GO:0017116 | single-stranded DNA-dependent ATP-dependent DNA helicase activity | | 10/553 | 20/17697 | 1.14E-10 | 1.06E-08 | 9.72E-09 | 10 |  |
| GO:0043142 | single-stranded DNA-dependent ATPase activity | | 10/553 | 20/17697 | 1.14E-10 | 1.06E-08 | 9.72E-09 | 10 |  |
| GO:0004003 | ATP-dependent DNA helicase activity | | 10/553 | 21/17697 | 2.12E-10 | 1.38E-08 | 1.26E-08 | 10 |  |
| GO:0008026 | ATP-dependent helicase activity | | 10/553 | 21/17697 | 2.12E-10 | 1.38E-08 | 1.26E-08 | 10 |  |
| GO:0070035 | purine NTP-dependent helicase activity | | 10/553 | 21/17697 | 2.12E-10 | 1.38E-08 | 1.26E-08 | 10 |  |
| GO:0043138 | 3'-5' DNA helicase activity | | 9/553 | 20/17697 | 3.29E-09 | 1.94E-07 | 1.78E-07 | 9 |  |
| GO:0016887 | ATPase activity | | 35/553 | 384/17697 | 1.56E-08 | 8.45E-07 | 7.76E-07 | 35 |  |
| GO:0008094 | DNA-dependent ATPase activity | | 13/553 | 69/17697 | 1.83E-07 | 8.70E-06 | 7.99E-06 | 13 |  |
| GO:0004386 | helicase activity | | 20/553 | 163/17697 | 1.88E-07 | 8.70E-06 | 7.99E-06 | 20 |  |
| GO:0035173 | histone kinase activity | | 7/553 | 17/17697 | 4.15E-07 | 1.80E-05 | 1.65E-05 | 7 |  |
| GO:0003777 | microtubule motor activity | | 13/553 | 84/17697 | 1.93E-06 | 7.83E-05 | 7.19E-05 | 13 |  |
| GO:0042623 | ATPase activity, coupled | | 25/553 | 284/17697 | 3.39E-06 | 0.00012927 | 0.00011867 | 25 |  |
| GO:0003774 | motor activity | | 14/553 | 136/17697 | 9.25E-05 | 0.00333554 | 0.00306207 | 14 |  |
| GO:0004674 | protein serine/threonine kinase activity | | 29/553 | 439/17697 | 0.00013171 | 0.00449907 | 0.0041302 | 29 |  |
| GO:0003697 | single-stranded DNA binding | | 12/553 | 113/17697 | 0.00021578 | 0.00700205 | 0.00642797 | 12 |  |
| GO:0042393 | histone binding | | 16/553 | 197/17697 | 0.00047795 | 0.0144208 | 0.01323846 | 16 |  |
| GO:0008187 | poly-pyrimidine tract binding | | 6/553 | 33/17697 | 0.00048884 | 0.0144208 | 0.01323846 | 6 |  |
| GO:0051371 | muscle alpha-actinin binding | | 5/553 | 23/17697 | 0.00061706 | 0.01741183 | 0.01598426 | 5 |  |
| GO:0016538 | cyclin-dependent protein serine/threonine kinase regulator activity | | 7/553 | 49/17697 | 0.0007704 | 0.02083298 | 0.01912491 | 7 |  |
| GO:0044325 | ion channel binding | | 11/553 | 124/17697 | 0.00175967 | 0.04568115 | 0.04193582 | 11 |  |
| GO:0005201 | extracellular matrix structural constituent | | 13/553 | 163/17697 | 0.00185408 | 0.04628057 | 0.0424861 | 13 |  |
| GO:0004712 | protein serine/threonine/tyrosine kinase activity | | 6/553 | 43/17697 | 0.00206695 | 0.04884347 | 0.04483887 | 6 |  |
| GO:0008266 | poly(U) RNA binding | | 5/553 | 30/17697 | 0.00218253 | 0.04884347 | 0.04483887 | 5 |  |
| GO:0070717 | poly-purine tract binding | | 5/553 | 30/17697 | 0.00218253 | 0.04884347 | 0.04483887 | 5 |  |
| GO:0008022 | protein C-terminus binding | | 14/553 | 187/17697 | 0.0022839 | 0.04918415 | 0.04515162 | 14 |  |
| GO:0003779 | actin binding | | 25/553 | 431/17697 | 0.00234932 | 0.04918415 | 0.04515162 | 25 |  |
