# Supplementary material for: POC1A acts as a promising prognostic biomarker associated with high tumor immune cell infiltration in gastric cancer
Source: Aging (Albany NY). 2020 Oct 14;12(19):18982–9011. doi: 10.18632/aging.103624 (PMC7732308; doi:10.18632/aging.103624)
Supplement: Supplementary File 7 [file aging-12-103624-s004..docx]

**Supplementary Table 7** Genomic alteration genes of POC1A in STAD

| **Gene** | **Cytoband** | **Alteration** | **Altered group** | **Unaltered group** | **Log Ratio** | **p-Value** | **q-Value** | **Enriched in** |
| --- | --- | --- | --- | --- | --- | --- | --- | --- |
| ALAS1 | 3p21.2 | DeepDel | 7 (25.93%) | 0 (0.00%) | >10 | 8.20E-10 | 4.46E-06 | Altered group |
| PPM1M | 3p21.2 | DeepDel | 7 (25.93%) | 0 (0.00%) | >10 | 8.20E-10 | 4.46E-06 | Altered group |
| PRKCD | 3p21.1 | DeepDel | 7 (25.93%) | 0 (0.00%) | >10 | 8.20E-10 | 4.46E-06 | Altered group |
| RFT1 | 3p21.1 | DeepDel | 7 (25.93%) | 0 (0.00%) | >10 | 8.20E-10 | 4.46E-06 | Altered group |
| TKT | 3p21.1 | DeepDel | 7 (25.93%) | 0 (0.00%) | >10 | 8.20E-10 | 4.46E-06 | Altered group |
| TLR9 | 3p21.2 | DeepDel | 7 (25.93%) | 0 (0.00%) | >10 | 8.20E-10 | 4.46E-06 | Altered group |
| TWF2 | 3p21.2 | DeepDel | 7 (25.93%) | 0 (0.00%) | >10 | 8.20E-10 | 4.46E-06 | Altered group |
| BAP1 | 3p21.1 | DeepDel | 7 (25.93%) | 1 (0.24%) | 6.75 | 1.11E-08 | 1.40E-05 | Altered group |
| DNAH1 | 3p21.1 | DeepDel | 7 (25.93%) | 1 (0.24%) | 6.75 | 1.11E-08 | 1.40E-05 | Altered group |
| GLYCTK | 3p21.2 | DeepDel | 7 (25.93%) | 1 (0.24%) | 6.75 | 1.11E-08 | 1.40E-05 | Altered group |
| GNL3 | 3p21.1 | DeepDel | 7 (25.93%) | 1 (0.24%) | 6.75 | 1.11E-08 | 1.40E-05 | Altered group |
| NISCH | 3p21.1 | DeepDel | 7 (25.93%) | 1 (0.24%) | 6.75 | 1.11E-08 | 1.40E-05 | Altered group |
| NT5DC2 | 3p21.1 | DeepDel | 7 (25.93%) | 1 (0.24%) | 6.75 | 1.11E-08 | 1.40E-05 | Altered group |
| PHF7 | 3p21.1 | DeepDel | 7 (25.93%) | 1 (0.24%) | 6.75 | 1.11E-08 | 1.40E-05 | Altered group |
| RNU6ATAC16P | 3p21.1 | DeepDel | 7 (25.93%) | 1 (0.24%) | 6.75 | 1.11E-08 | 1.40E-05 | Altered group |
| SEMA3G | 3p21.1 | DeepDel | 7 (25.93%) | 1 (0.24%) | 6.75 | 1.11E-08 | 1.40E-05 | Altered group |
| SFMBT1 | 3p21.1 | DeepDel | 7 (25.93%) | 1 (0.24%) | 6.75 | 1.11E-08 | 1.40E-05 | Altered group |
| SMIM4 | 3p21.1 | DeepDel | 7 (25.93%) | 1 (0.24%) | 6.75 | 1.11E-08 | 1.40E-05 | Altered group |
| SNORD19 | 3p21.1 | DeepDel | 7 (25.93%) | 1 (0.24%) | 6.75 | 1.11E-08 | 1.40E-05 | Altered group |
| SNORD19B | 3p21.1 | DeepDel | 7 (25.93%) | 1 (0.24%) | 6.75 | 1.11E-08 | 1.40E-05 | Altered group |
| SNORD69 | 3p21.1 | DeepDel | 7 (25.93%) | 1 (0.24%) | 6.75 | 1.11E-08 | 1.40E-05 | Altered group |
| STAB1 | 3p21.1 | DeepDel | 7 (25.93%) | 1 (0.24%) | 6.75 | 1.11E-08 | 1.40E-05 | Altered group |
| TNNC1 | 3p21.1 | DeepDel | 7 (25.93%) | 1 (0.24%) | 6.75 | 1.11E-08 | 1.40E-05 | Altered group |
| WDR82 | 3p21.2 | DeepDel | 7 (25.93%) | 1 (0.24%) | 6.75 | 1.11E-08 | 1.40E-05 | Altered group |
| ARIH2OS | 3p21.31 | DeepDel | 6 (22.22%) | 0 (0.00%) | >10 | 1.84E-08 | 1.40E-05 | Altered group |
| CELSR3 | 3p21.31 | DeepDel | 6 (22.22%) | 0 (0.00%) | >10 | 1.84E-08 | 1.40E-05 | Altered group |
| COL7A1 | 3p21.31 | DeepDel | 6 (22.22%) | 0 (0.00%) | >10 | 1.84E-08 | 1.40E-05 | Altered group |
| DCAF1 | 3p21.2 | DeepDel | 6 (22.22%) | 0 (0.00%) | >10 | 1.84E-08 | 1.40E-05 | Altered group |
| DCP1A | 3p21.1 | DeepDel | 6 (22.22%) | 0 (0.00%) | >10 | 1.84E-08 | 1.40E-05 | Altered group |
| DUSP7 | 3p21.2 | DeepDel | 6 (22.22%) | 0 (0.00%) | >10 | 1.84E-08 | 1.40E-05 | Altered group |
| GPR62 | 3p21.2 | DeepDel | 6 (22.22%) | 0 (0.00%) | >10 | 1.84E-08 | 1.40E-05 | Altered group |
| GRM2 | 3p21.2 | DeepDel | 6 (22.22%) | 0 (0.00%) | >10 | 1.84E-08 | 1.40E-05 | Altered group |
| IQCF1 | 3p21.2 | DeepDel | 6 (22.22%) | 0 (0.00%) | >10 | 1.84E-08 | 1.40E-05 | Altered group |
| IQCF2 | 3p21.2 | DeepDel | 6 (22.22%) | 0 (0.00%) | >10 | 1.84E-08 | 1.40E-05 | Altered group |
| IQCF3 | 3p21.2 | DeepDel | 6 (22.22%) | 0 (0.00%) | >10 | 1.84E-08 | 1.40E-05 | Altered group |
| IQCF5 | 3p21.2 | DeepDel | 6 (22.22%) | 0 (0.00%) | >10 | 1.84E-08 | 1.40E-05 | Altered group |
| IQCF6 | 3p21.2 | DeepDel | 6 (22.22%) | 0 (0.00%) | >10 | 1.84E-08 | 1.40E-05 | Altered group |
| LINC00696 | 3p21.2 | DeepDel | 6 (22.22%) | 0 (0.00%) | >10 | 1.84E-08 | 1.40E-05 | Altered group |
| MIR-711/711 |  | DeepDel | 6 (22.22%) | 0 (0.00%) | >10 | 1.84E-08 | 1.40E-05 | Altered group |
| PARP3 | 3p21.2 | DeepDel | 6 (22.22%) | 0 (0.00%) | >10 | 1.84E-08 | 1.40E-05 | Altered group |
| PFKFB4 | 3p21.31 | DeepDel | 6 (22.22%) | 0 (0.00%) | >10 | 1.84E-08 | 1.40E-05 | Altered group |
| RN7SL504P | 3p21.2 | DeepDel | 6 (22.22%) | 0 (0.00%) | >10 | 1.84E-08 | 1.40E-05 | Altered group |
| RNA5SP132 | 3p21.2 | DeepDel | 6 (22.22%) | 0 (0.00%) | >10 | 1.84E-08 | 1.40E-05 | Altered group |
| RRP9 | 3p21.2 | DeepDel | 6 (22.22%) | 0 (0.00%) | >10 | 1.84E-08 | 1.40E-05 | Altered group |
| SLC25A20 | 3p21.31 | DeepDel | 6 (22.22%) | 0 (0.00%) | >10 | 1.84E-08 | 1.40E-05 | Altered group |
| SLC26A6 | 3p21.31 | DeepDel | 6 (22.22%) | 0 (0.00%) | >10 | 1.84E-08 | 1.40E-05 | Altered group |
| TEX264 | 3p21.2 | DeepDel | 6 (22.22%) | 0 (0.00%) | >10 | 1.84E-08 | 1.40E-05 | Altered group |
| TMEM89 | 3p21.31 | DeepDel | 6 (22.22%) | 0 (0.00%) | >10 | 1.84E-08 | 1.40E-05 | Altered group |
| UCN2 | 3p21.31 | DeepDel | 6 (22.22%) | 0 (0.00%) | >10 | 1.84E-08 | 1.40E-05 | Altered group |
| UQCRC1 | 3p21.31 | DeepDel | 6 (22.22%) | 0 (0.00%) | >10 | 1.84E-08 | 1.40E-05 | Altered group |
| GLT8D1 | 3p21.1 | DeepDel | 7 (25.93%) | 2 (0.48%) | 5.75 | 4.80E-08 | 3.09E-05 | Altered group |
| ITIH1 | 3p21.1 | DeepDel | 7 (25.93%) | 2 (0.48%) | 5.75 | 4.80E-08 | 3.09E-05 | Altered group |
| ITIH3 | 3p21.1 | DeepDel | 7 (25.93%) | 2 (0.48%) | 5.75 | 4.80E-08 | 3.09E-05 | Altered group |
| ITIH4 | 3p21.1 | DeepDel | 7 (25.93%) | 2 (0.48%) | 5.75 | 4.80E-08 | 3.09E-05 | Altered group |
| MUSTN1 | 3p21.1 | DeepDel | 7 (25.93%) | 2 (0.48%) | 5.75 | 4.80E-08 | 3.09E-05 | Altered group |
| NEK4 | 3p21.1 | DeepDel | 7 (25.93%) | 2 (0.48%) | 5.75 | 4.80E-08 | 3.09E-05 | Altered group |
| PBRM1 | 3p21.1 | DeepDel | 7 (25.93%) | 2 (0.48%) | 5.75 | 4.80E-08 | 3.09E-05 | Altered group |
| SPCS1 | 3p21.1 | DeepDel | 7 (25.93%) | 2 (0.48%) | 5.75 | 4.80E-08 | 3.09E-05 | Altered group |
| STIMATE | 3p21.1 | DeepDel | 7 (25.93%) | 2 (0.48%) | 5.75 | 4.80E-08 | 3.09E-05 | Altered group |
| ABHD14A | 3p21.2 | DeepDel | 6 (22.22%) | 1 (0.24%) | 6.52 | 2.01E-07 | 6.83E-05 | Altered group |
| ABHD14B | 3p21.2 | DeepDel | 6 (22.22%) | 1 (0.24%) | 6.52 | 2.01E-07 | 6.83E-05 | Altered group |
| ACY1 | 3p21.2 | DeepDel | 6 (22.22%) | 1 (0.24%) | 6.52 | 2.01E-07 | 6.83E-05 | Altered group |
| ARIH2 | 3p21.31 | DeepDel | 6 (22.22%) | 1 (0.24%) | 6.52 | 2.01E-07 | 6.83E-05 | Altered group |
| C3ORF18 | 3p21.31 | DeepDel | 6 (22.22%) | 1 (0.24%) | 6.52 | 2.01E-07 | 6.83E-05 | Altered group |
| C3ORF62 | 3p21.31 | DeepDel | 6 (22.22%) | 1 (0.24%) | 6.52 | 2.01E-07 | 6.83E-05 | Altered group |
| CAMKV | 3p21.31 | DeepDel | 6 (22.22%) | 1 (0.24%) | 6.52 | 2.01E-07 | 6.83E-05 | Altered group |
| CCDC36 | 3p21.31 | DeepDel | 6 (22.22%) | 1 (0.24%) | 6.52 | 2.01E-07 | 6.83E-05 | Altered group |
| CDHR4 | 3p21.31 | DeepDel | 6 (22.22%) | 1 (0.24%) | 6.52 | 2.01E-07 | 6.83E-05 | Altered group |
| CISH | 3p21.2 | DeepDel | 6 (22.22%) | 1 (0.24%) | 6.52 | 2.01E-07 | 6.83E-05 | Altered group |
| DAG1 | 3p21.31 | DeepDel | 6 (22.22%) | 1 (0.24%) | 6.52 | 2.01E-07 | 6.83E-05 | Altered group |
| DALRD3 | 3p21.31 | DeepDel | 6 (22.22%) | 1 (0.24%) | 6.52 | 2.01E-07 | 6.83E-05 | Altered group |
| GNAI2 | 3p21.31 | DeepDel | 6 (22.22%) | 1 (0.24%) | 6.52 | 2.01E-07 | 6.83E-05 | Altered group |
| GNAT1 | 3p21.31 | DeepDel | 6 (22.22%) | 1 (0.24%) | 6.52 | 2.01E-07 | 6.83E-05 | Altered group |
| GPX1 | 3p21.31 | DeepDel | 6 (22.22%) | 1 (0.24%) | 6.52 | 2.01E-07 | 6.83E-05 | Altered group |
| HEMK1 | 3p21.31 | DeepDel | 6 (22.22%) | 1 (0.24%) | 6.52 | 2.01E-07 | 6.83E-05 | Altered group |
| HYAL1 | 3p21.31 | DeepDel | 6 (22.22%) | 1 (0.24%) | 6.52 | 2.01E-07 | 6.83E-05 | Altered group |
| HYAL2 | 3p21.31 | DeepDel | 6 (22.22%) | 1 (0.24%) | 6.52 | 2.01E-07 | 6.83E-05 | Altered group |
| HYAL3 | 3p21.31 | DeepDel | 6 (22.22%) | 1 (0.24%) | 6.52 | 2.01E-07 | 6.83E-05 | Altered group |
| IFRD2 | 3p21.31 | DeepDel | 6 (22.22%) | 1 (0.24%) | 6.52 | 2.01E-07 | 6.83E-05 | Altered group |
| IMPDH2 | 3p21.31 | DeepDel | 6 (22.22%) | 1 (0.24%) | 6.52 | 2.01E-07 | 6.83E-05 | Altered group |
| INKA1 | 3p21.31 | DeepDel | 6 (22.22%) | 1 (0.24%) | 6.52 | 2.01E-07 | 6.83E-05 | Altered group |
| IP6K1 | 3p21.31 | DeepDel | 6 (22.22%) | 1 (0.24%) | 6.52 | 2.01E-07 | 6.83E-05 | Altered group |
| IP6K2 | 3p21.31 | DeepDel | 6 (22.22%) | 1 (0.24%) | 6.52 | 2.01E-07 | 6.83E-05 | Altered group |
| LSMEM2 | 3p21.31 | DeepDel | 6 (22.22%) | 1 (0.24%) | 6.52 | 2.01E-07 | 6.83E-05 | Altered group |
| MAPKAPK3 | 3p21.2 | DeepDel | 6 (22.22%) | 1 (0.24%) | 6.52 | 2.01E-07 | 6.83E-05 | Altered group |
| MIR-4271/4271 | | DeepDel | 6 (22.22%) | 1 (0.24%) | 6.52 | 2.01E-07 | 6.83E-05 | Altered group |
| MIR-566/566 |  | DeepDel | 6 (22.22%) | 1 (0.24%) | 6.52 | 2.01E-07 | 6.83E-05 | Altered group |
| NAA80 | 3p21.31 | DeepDel | 6 (22.22%) | 1 (0.24%) | 6.52 | 2.01E-07 | 6.83E-05 | Altered group |
| NCKIPSD | 3p21.31 | DeepDel | 6 (22.22%) | 1 (0.24%) | 6.52 | 2.01E-07 | 6.83E-05 | Altered group |
| NDUFAF3 | 3p21.31 | DeepDel | 6 (22.22%) | 1 (0.24%) | 6.52 | 2.01E-07 | 6.83E-05 | Altered group |
| P4HTM | 3p21.31\|3p21.3 | DeepDel | 6 (22.22%) | 1 (0.24%) | 6.52 | 2.01E-07 | 6.83E-05 | Altered group |
| PCBP4 | 3p21.2 | DeepDel | 6 (22.22%) | 1 (0.24%) | 6.52 | 2.01E-07 | 6.83E-05 | Altered group |
| PRKAR2A | 3p21.31 | DeepDel | 6 (22.22%) | 1 (0.24%) | 6.52 | 2.01E-07 | 6.83E-05 | Altered group |
| QARS | 3p21.31 | DeepDel | 6 (22.22%) | 1 (0.24%) | 6.52 | 2.01E-07 | 6.83E-05 | Altered group |
| QRICH1 | 3p21.31 | DeepDel | 6 (22.22%) | 1 (0.24%) | 6.52 | 2.01E-07 | 6.83E-05 | Altered group |
| RAD54L2 | 3p21.2 | DeepDel | 6 (22.22%) | 1 (0.24%) | 6.52 | 2.01E-07 | 6.83E-05 | Altered group |
| RBM5 | 3p21.31 | DeepDel | 6 (22.22%) | 1 (0.24%) | 6.52 | 2.01E-07 | 6.83E-05 | Altered group |
| RN7SL182P | 3p21.31 | DeepDel | 6 (22.22%) | 1 (0.24%) | 6.52 | 2.01E-07 | 6.83E-05 | Altered group |
| RN7SL217P | 3p21.31 | DeepDel | 6 (22.22%) | 1 (0.24%) | 6.52 | 2.01E-07 | 6.83E-05 | Altered group |
| RNA5SP130 | 3p21.31 | DeepDel | 6 (22.22%) | 1 (0.24%) | 6.52 | 2.01E-07 | 6.83E-05 | Altered group |
| RNU6ATAC29P | 3p21.2 | DeepDel | 6 (22.22%) | 1 (0.24%) | 6.52 | 2.01E-07 | 6.83E-05 | Altered group |
| RPL29 | 3p21.2 | DeepDel | 6 (22.22%) | 1 (0.24%) | 6.52 | 2.01E-07 | 6.83E-05 | Altered group |
| SEMA3B | 3p21.31 | DeepDel | 6 (22.22%) | 1 (0.24%) | 6.52 | 2.01E-07 | 6.83E-05 | Altered group |
| SEMA3F | 3p21.31 | DeepDel | 6 (22.22%) | 1 (0.24%) | 6.52 | 2.01E-07 | 6.83E-05 | Altered group |
| SLC38A3 | 3p21.31 | DeepDel | 6 (22.22%) | 1 (0.24%) | 6.52 | 2.01E-07 | 6.83E-05 | Altered group |
| SNORD63 | 5q31.2 | DeepDel | 6 (22.22%) | 1 (0.24%) | 6.52 | 2.01E-07 | 6.83E-05 | Altered group |
| TRAIP | 3p21.31 | DeepDel | 6 (22.22%) | 1 (0.24%) | 6.52 | 2.01E-07 | 6.83E-05 | Altered group |
| TUSC2 | 3p21.31 | DeepDel | 6 (22.22%) | 1 (0.24%) | 6.52 | 2.01E-07 | 6.83E-05 | Altered group |
| UBA7 | 3p21.31 | DeepDel | 6 (22.22%) | 1 (0.24%) | 6.52 | 2.01E-07 | 6.83E-05 | Altered group |
| USP19 | 3p21.31 | DeepDel | 6 (22.22%) | 1 (0.24%) | 6.52 | 2.01E-07 | 6.83E-05 | Altered group |
| USP4 | 3p21.31 | DeepDel | 6 (22.22%) | 1 (0.24%) | 6.52 | 2.01E-07 | 6.83E-05 | Altered group |
| WDR6 | 3p21.31 | DeepDel | 6 (22.22%) | 1 (0.24%) | 6.52 | 2.01E-07 | 6.83E-05 | Altered group |
| APPL1 | 3p14.3 | DeepDel | 5 (18.52%) | 0 (0.00%) | >10 | 3.96E-07 | 1.06E-04 | Altered group |
| ARF4 | 3p14.3 | DeepDel | 5 (18.52%) | 0 (0.00%) | >10 | 3.96E-07 | 1.06E-04 | Altered group |
| ASB14 | 3p14.3 | DeepDel | 5 (18.52%) | 0 (0.00%) | >10 | 3.96E-07 | 1.06E-04 | Altered group |
| ATRIP | 3p21.31 | DeepDel | 5 (18.52%) | 0 (0.00%) | >10 | 3.96E-07 | 1.06E-04 | Altered group |
| CAMP | 3p21.31 | DeepDel | 5 (18.52%) | 0 (0.00%) | >10 | 3.96E-07 | 1.06E-04 | Altered group |
| CCDC51 | 3p21.31 | DeepDel | 5 (18.52%) | 0 (0.00%) | >10 | 3.96E-07 | 1.06E-04 | Altered group |
| CCDC66 | 3p14.3 | DeepDel | 5 (18.52%) | 0 (0.00%) | >10 | 3.96E-07 | 1.06E-04 | Altered group |
| CDC25A | 3p21.31 | DeepDel | 5 (18.52%) | 0 (0.00%) | >10 | 3.96E-07 | 1.06E-04 | Altered group |
| DNAH12 | 3p14.3 | DeepDel | 5 (18.52%) | 0 (0.00%) | >10 | 3.96E-07 | 1.06E-04 | Altered group |
| ESRG | 3p14.3\|3p14.3 | DeepDel | 5 (18.52%) | 0 (0.00%) | >10 | 3.96E-07 | 1.06E-04 | Altered group |
| FBXW12 | 3p21.31 | DeepDel | 5 (18.52%) | 0 (0.00%) | >10 | 3.96E-07 | 1.06E-04 | Altered group |
| HESX1 | 3p14.3 | DeepDel | 5 (18.52%) | 0 (0.00%) | >10 | 3.96E-07 | 1.06E-04 | Altered group |
| IL17RD | 3p14.3 | DeepDel | 5 (18.52%) | 0 (0.00%) | >10 | 3.96E-07 | 1.06E-04 | Altered group |
| MANF | 3p21.2 | DeepDel | 5 (18.52%) | 0 (0.00%) | >10 | 3.96E-07 | 1.06E-04 | Altered group |
| MIR-4443/4443 | | DeepDel | 5 (18.52%) | 0 (0.00%) | >10 | 3.96E-07 | 1.06E-04 | Altered group |
| NME6 | 3p21.31 | DeepDel | 5 (18.52%) | 0 (0.00%) | >10 | 3.96E-07 | 1.06E-04 | Altered group |
| PDE12 | 3p14.3 | DeepDel | 5 (18.52%) | 0 (0.00%) | >10 | 3.96E-07 | 1.06E-04 | Altered group |
| PLXNB1 | 3p21.31 | DeepDel | 5 (18.52%) | 0 (0.00%) | >10 | 3.96E-07 | 1.06E-04 | Altered group |
| RBM15B | 3p21.2 | DeepDel | 5 (18.52%) | 0 (0.00%) | >10 | 3.96E-07 | 1.06E-04 | Altered group |
| RN7SKP45 | 3p14.3 | DeepDel | 5 (18.52%) | 0 (0.00%) | >10 | 3.96E-07 | 1.06E-04 | Altered group |
| RN7SL321P | 3p21.31 | DeepDel | 5 (18.52%) | 0 (0.00%) | >10 | 3.96E-07 | 1.06E-04 | Altered group |
| RNA5SP133 | 3p14.3 | DeepDel | 5 (18.52%) | 0 (0.00%) | >10 | 3.96E-07 | 1.06E-04 | Altered group |
| RNU6ATAC26P | 3p14.3 | DeepDel | 5 (18.52%) | 0 (0.00%) | >10 | 3.96E-07 | 1.06E-04 | Altered group |
| SELENOK | 3p21.1 | DeepDel | 5 (18.52%) | 0 (0.00%) | >10 | 3.96E-07 | 1.06E-04 | Altered group |
| SHISA5 | 3p21.31 | DeepDel | 5 (18.52%) | 0 (0.00%) | >10 | 3.96E-07 | 1.06E-04 | Altered group |
| SPINK8 | 3p21.31 | DeepDel | 5 (18.52%) | 0 (0.00%) | >10 | 3.96E-07 | 1.06E-04 | Altered group |
| TASOR | 3p14.3 | DeepDel | 5 (18.52%) | 0 (0.00%) | >10 | 3.96E-07 | 1.06E-04 | Altered group |
| TMA7 | 3p21.31 | DeepDel | 5 (18.52%) | 0 (0.00%) | >10 | 3.96E-07 | 1.06E-04 | Altered group |
| TREX1 | 3p21.31 | DeepDel | 5 (18.52%) | 0 (0.00%) | >10 | 3.96E-07 | 1.06E-04 | Altered group |
| ZNF589 | 3p21.31 | DeepDel | 5 (18.52%) | 0 (0.00%) | >10 | 3.96E-07 | 1.06E-04 | Altered group |
| AMIGO3 | 3p21.31 | DeepDel | 6 (22.22%) | 2 (0.48%) | 5.52 | 7.71E-07 | 1.81E-04 | Altered group |
| APEH | 3p21.31 | DeepDel | 6 (22.22%) | 2 (0.48%) | 5.52 | 7.71E-07 | 1.81E-04 | Altered group |
| BSN | 3p21.31 | DeepDel | 6 (22.22%) | 2 (0.48%) | 5.52 | 7.71E-07 | 1.81E-04 | Altered group |
| C3ORF84 | 3p21.31 | DeepDel | 6 (22.22%) | 2 (0.48%) | 5.52 | 7.71E-07 | 1.81E-04 | Altered group |
| CACNA1D | 3p21.1 | DeepDel | 6 (22.22%) | 2 (0.48%) | 5.52 | 7.71E-07 | 1.81E-04 | Altered group |
| CACNA2D2 | 3p21.31 | DeepDel | 6 (22.22%) | 2 (0.48%) | 5.52 | 7.71E-07 | 1.81E-04 | Altered group |
| CCDC71 | 3p21.31 | DeepDel | 6 (22.22%) | 2 (0.48%) | 5.52 | 7.71E-07 | 1.81E-04 | Altered group |
| CYB561D2 | 3p21.31 | DeepDel | 6 (22.22%) | 2 (0.48%) | 5.52 | 7.71E-07 | 1.81E-04 | Altered group |
| DOCK3 | 3p21.2 | DeepDel | 6 (22.22%) | 2 (0.48%) | 5.52 | 7.71E-07 | 1.81E-04 | Altered group |
| GMPPB | 3p21.31 | DeepDel | 6 (22.22%) | 2 (0.48%) | 5.52 | 7.71E-07 | 1.81E-04 | Altered group |
| KLHDC8B | 3p21.31 | DeepDel | 6 (22.22%) | 2 (0.48%) | 5.52 | 7.71E-07 | 1.81E-04 | Altered group |
| LAMB2 | 3p21.31 | DeepDel | 6 (22.22%) | 2 (0.48%) | 5.52 | 7.71E-07 | 1.81E-04 | Altered group |
| MST1 | 3p21.31 | DeepDel | 6 (22.22%) | 2 (0.48%) | 5.52 | 7.71E-07 | 1.81E-04 | Altered group |
| MST1R | 3p21.31 | DeepDel | 6 (22.22%) | 2 (0.48%) | 5.52 | 7.71E-07 | 1.81E-04 | Altered group |
| NPRL2 | 3p21.31 | DeepDel | 6 (22.22%) | 2 (0.48%) | 5.52 | 7.71E-07 | 1.81E-04 | Altered group |
| RASSF1 | 3p21.31 | DeepDel | 6 (22.22%) | 2 (0.48%) | 5.52 | 7.71E-07 | 1.81E-04 | Altered group |
| RNA5SP131 | 3p21.31 | DeepDel | 6 (22.22%) | 2 (0.48%) | 5.52 | 7.71E-07 | 1.81E-04 | Altered group |
| RNF123 | 3p21.31 | DeepDel | 6 (22.22%) | 2 (0.48%) | 5.52 | 7.71E-07 | 1.81E-04 | Altered group |
| TMEM115 | 3p21.31 | DeepDel | 6 (22.22%) | 2 (0.48%) | 5.52 | 7.71E-07 | 1.81E-04 | Altered group |
| ZMYND10 | 3p21.31 | DeepDel | 6 (22.22%) | 2 (0.48%) | 5.52 | 7.71E-07 | 1.81E-04 | Altered group |
| AMT | 3p21.31 | DeepDel | 6 (22.22%) | 3 (0.72%) | 4.94 | 2.22E-06 | 4.99E-04 | Altered group |
| MON1A | 3p21.31 | DeepDel | 6 (22.22%) | 3 (0.72%) | 4.94 | 2.22E-06 | 4.99E-04 | Altered group |
| NICN1 | 3p21.31 | DeepDel | 6 (22.22%) | 3 (0.72%) | 4.94 | 2.22E-06 | 4.99E-04 | Altered group |
| RBM6 | 3p21.31 | DeepDel | 6 (22.22%) | 3 (0.72%) | 4.94 | 2.22E-06 | 4.99E-04 | Altered group |
| RHOA | 3p21.31 | DeepDel | 6 (22.22%) | 3 (0.72%) | 4.94 | 2.22E-06 | 4.99E-04 | Altered group |
| SLMAP | 3p14.3 | DeepDel | 6 (22.22%) | 3 (0.72%) | 4.94 | 2.22E-06 | 4.99E-04 | Altered group |
| TCTA | 3p21.31 | DeepDel | 6 (22.22%) | 3 (0.72%) | 4.94 | 2.22E-06 | 4.99E-04 | Altered group |
| ACTR8 | 3p21.1 | DeepDel | 5 (18.52%) | 1 (0.24%) | 6.26 | 3.42E-06 | 7.34E-04 | Altered group |
| ARHGEF3 | 3p14.3 | DeepDel | 5 (18.52%) | 1 (0.24%) | 6.26 | 3.42E-06 | 7.34E-04 | Altered group |
| CHDH | 3p21.1 | DeepDel | 5 (18.52%) | 1 (0.24%) | 6.26 | 3.42E-06 | 7.34E-04 | Altered group |
| DENND6A | 3p14.3 | DeepDel | 5 (18.52%) | 1 (0.24%) | 6.26 | 3.42E-06 | 7.34E-04 | Altered group |
| LRTM1 | 3p14.3 | DeepDel | 5 (18.52%) | 1 (0.24%) | 6.26 | 3.42E-06 | 7.34E-04 | Altered group |
| MIR-3938/3938 | | DeepDel | 5 (18.52%) | 1 (0.24%) | 6.26 | 3.42E-06 | 7.34E-04 | Altered group |
| SPATA12 | 3p14.3 | DeepDel | 5 (18.52%) | 1 (0.24%) | 6.26 | 3.42E-06 | 7.34E-04 | Altered group |
| WNT5A | 3p14.3 | DeepDel | 5 (18.52%) | 1 (0.24%) | 6.26 | 3.42E-06 | 7.34E-04 | Altered group |
| ERC2 | 3p14.3 | DeepDel | 6 (22.22%) | 4 (0.97%) | 4.52 | 5.32E-06 | 1.14E-03 | Altered group |
| ABHD6 | 3p14.3 | DeepDel | 4 (14.81%) | 0 (0.00%) | >10 | 8.17E-06 | 1.65E-03 | Altered group |
| ACOX2 | 3p14.3 | DeepDel | 4 (14.81%) | 0 (0.00%) | >10 | 8.17E-06 | 1.65E-03 | Altered group |
| DNASE1L3 | 3p14.3 | DeepDel | 4 (14.81%) | 0 (0.00%) | >10 | 8.17E-06 | 1.65E-03 | Altered group |
| FAM107A | 3p14.3-p14.2 | DeepDel | 4 (14.81%) | 0 (0.00%) | >10 | 8.17E-06 | 1.65E-03 | Altered group |
| FAM3D | 3p14.2 | DeepDel | 4 (14.81%) | 0 (0.00%) | >10 | 8.17E-06 | 1.65E-03 | Altered group |
| KCTD6 | 3p14.3 | DeepDel | 4 (14.81%) | 0 (0.00%) | >10 | 8.17E-06 | 1.65E-03 | Altered group |
| PDHB | 3p14.3 | DeepDel | 4 (14.81%) | 0 (0.00%) | >10 | 8.17E-06 | 1.65E-03 | Altered group |
| PXK | 3p14.3 | DeepDel | 4 (14.81%) | 0 (0.00%) | >10 | 8.17E-06 | 1.65E-03 | Altered group |
| RN7SL664P | 3p21.31 | DeepDel | 4 (14.81%) | 0 (0.00%) | >10 | 8.17E-06 | 1.65E-03 | Altered group |
| RPP14 | 3p14.3 | DeepDel | 4 (14.81%) | 0 (0.00%) | >10 | 8.17E-06 | 1.65E-03 | Altered group |
| CACNA2D3 | 3p21.1-p14.3 | DeepDel | 5 (18.52%) | 2 (0.48%) | 5.26 | 1.15E-05 | 2.29E-03 | Altered group |
| IL17RB | 3p21.1 | DeepDel | 5 (18.52%) | 2 (0.48%) | 5.26 | 1.15E-05 | 2.29E-03 | Altered group |
| ALS2CL | 3p21.31 | DeepDel | 4 (14.81%) | 1 (0.24%) | 5.94 | 5.41E-05 | 9.70E-03 | Altered group |
| C3ORF67 | 3p14.2 | DeepDel | 4 (14.81%) | 1 (0.24%) | 5.94 | 5.41E-05 | 9.70E-03 | Altered group |
| CSPG5 | 3p21.31 | DeepDel | 4 (14.81%) | 1 (0.24%) | 5.94 | 5.41E-05 | 9.70E-03 | Altered group |
| ELP6 | 3p21.31 | DeepDel | 4 (14.81%) | 1 (0.24%) | 5.94 | 5.41E-05 | 9.70E-03 | Altered group |
| KIF9 | 3p21.31 | DeepDel | 4 (14.81%) | 1 (0.24%) | 5.94 | 5.41E-05 | 9.70E-03 | Altered group |
| KLHL18 | 3p21.31 | DeepDel | 4 (14.81%) | 1 (0.24%) | 5.94 | 5.41E-05 | 9.70E-03 | Altered group |
| LRRC2 | 3p21.31 | DeepDel | 4 (14.81%) | 1 (0.24%) | 5.94 | 5.41E-05 | 9.70E-03 | Altered group |
| LRRC2-AS1 | 3p21.31 | DeepDel | 4 (14.81%) | 1 (0.24%) | 5.94 | 5.41E-05 | 9.70E-03 | Altered group |
| MYL3 | 3p21.31 | DeepDel | 4 (14.81%) | 1 (0.24%) | 5.94 | 5.41E-05 | 9.70E-03 | Altered group |
| PRSS42P | 3p21.31 | DeepDel | 4 (14.81%) | 1 (0.24%) | 5.94 | 5.41E-05 | 9.70E-03 | Altered group |
| PRSS44P | 3p21.31 | DeepDel | 4 (14.81%) | 1 (0.24%) | 5.94 | 5.41E-05 | 9.70E-03 | Altered group |
| PRSS45P | 3p21.31 | DeepDel | 4 (14.81%) | 1 (0.24%) | 5.94 | 5.41E-05 | 9.70E-03 | Altered group |
| PRSS46P | 3p21.31 | DeepDel | 4 (14.81%) | 1 (0.24%) | 5.94 | 5.41E-05 | 9.70E-03 | Altered group |
| PRSS50 | 3p21.31 | DeepDel | 4 (14.81%) | 1 (0.24%) | 5.94 | 5.41E-05 | 9.70E-03 | Altered group |
| PTH1R | 3p21.31 | DeepDel | 4 (14.81%) | 1 (0.24%) | 5.94 | 5.41E-05 | 9.70E-03 | Altered group |
| PTPN23 | 3p21.31 | DeepDel | 4 (14.81%) | 1 (0.24%) | 5.94 | 5.41E-05 | 9.70E-03 | Altered group |
| RN7SL870P | 3p21.31 | DeepDel | 4 (14.81%) | 1 (0.24%) | 5.94 | 5.41E-05 | 9.70E-03 | Altered group |
| SCAP | 3p21.31 | DeepDel | 4 (14.81%) | 1 (0.24%) | 5.94 | 5.41E-05 | 9.70E-03 | Altered group |
| SMARCC1 | 3p21.31 | DeepDel | 4 (14.81%) | 1 (0.24%) | 5.94 | 5.41E-05 | 9.70E-03 | Altered group |
| SNORD77 | 1q25.1 | DeepDel | 4 (14.81%) | 1 (0.24%) | 5.94 | 5.41E-05 | 9.70E-03 | Altered group |
| TDGF1 | 3p21.31 | DeepDel | 4 (14.81%) | 1 (0.24%) | 5.94 | 5.41E-05 | 9.70E-03 | Altered group |
| TMIE | 3p21.31 | DeepDel | 4 (14.81%) | 1 (0.24%) | 5.94 | 5.41E-05 | 9.70E-03 | Altered group |
