# Supplementary material for: POC1A acts as a promising prognostic biomarker associated with high tumor immune cell infiltration in gastric cancer
Source: Aging (Albany NY). 2020 Oct 14;12(19):18982–9011. doi: 10.18632/aging.103624 (PMC7732308; doi:10.18632/aging.103624)
Supplement: Supplementary Tables 8, 9 and 10 [file aging-12-103624-s005..pdf]

SUPPLEMENTARY TABLES

Supplementary Table 8. Significant KEGG pathways of alteration genes of POC1A.

ID	Description	GeneRatio	BgRatio	pvalue	p.adjust	qvalue	Count
hsa04260	Cardiac muscle contraction	6/73	86/8016	0.00012251	0.01296701	0.01246601	6
hsa05410	Hypertrophic cardiomyopathy (HCM)	6/73	90/8016	0.00015765	0.01296701	0.01246601	6
hsa05414	Dilated cardiomyopathy (DCM)	6/73	96/8016	0.00022486	0.01296701	0.01246601	6
hsa00260	Glycine, serine and threonine metabolism	4/73	40/8016	0.00045152	0.01952831	0.01877379	4
hsa00531	Glycosaminoglycan degradation	3/73	19/8016	0.00063237	0.02187986	0.02103448	3
hsa04360	Axon guidance	7/73	181/8016	0.00123623	0.03564476	0.03426755	7

Supplementary Table 9. Significant GO annotations of alteration genes of POC1A (Biological Process and Mollecular Functions).

ID	Description	GeneRatio	BgRatio	pvalue	p.adjust	qvalue	Count
GO:0030212	hyaluronan metabolic process	6/166	37/18670	8.35E-07	0.00263953	0.00228095	6
GO:0050919	negative chemotaxis	6/166	46/18670	3.15E-06	0.00497728	0.00430112	6
GO:0016049	cell growth	16/166	484/18670	6.90E-06	0.00726886	0.00628138	16
GO:0001558	regulation of cell growth	14/166	416/18670	2.18E-05	0.01721154	0.01487336	14
GO:0071493	cellular response to UV-B	3/166	8/18670	3.74E-05	0.02365151	0.02043846	3
GO:1903510	mucopolysaccharide metabolic process	7/166	112/18670	6.41E-05	0.03056102	0.02640931	7
GO:0050808	synapse organization	13/166	408/18670	7.53E-05	0.03056102	0.02640931	13
GO:0048843	negative regulation of axon extension involved in axon guidance	4/166	26/18670	7.73E-05	0.03056102	0.02640931	4
GO:1902668	negative regulation of axon guidance	4/166	28/18670	0.00010447	0.03669279	0.03170808	4
GO:0004415	hyalurononglucosaminidase activity	3/164	8/17697	4.23E-05	0.01355295	0.01137836	3
GO:0030215	semaphorin receptor binding	4/164	23/17697	5.49E-05	0.01355295	0.01137836	4
GO:0033906	hyaluronoglucuronidase activity	2/164	2/17697	8.54E-05	0.01405602	0.01180071	2
GO:0015929	hexosaminidase activity	3/164	15/17697	0.00032759	0.04045704	0.03396564	3
GO:0098882	structural constituent of presynaptic active zone	2/164	4/17697	0.00050593	0.04600134	0.03862035	2
GO:0038191	neuropilin binding	3/164	18/17697	0.00057562	0.04600134	0.03862035	3
GO:0000828	inositol hexakisphosphate kinase activity	2/164	5/17697	0.00083808	0.04600134	0.03862035	2
GO:0000832	inositol hexakisphosphate 5-kinase activity	2/164	5/17697	0.00083808	0.04600134	0.03862035	2
GO:0099181	structural constituent of presynapse	2/164	5/17697	0.00083808	0.04600134	0.03862035	2

Supplementary Table 10. Correlations between immune infiltration levels of different immune cells and copy number alteration (CNA) of POC1A.

Cancer	Immune Cells	CNA	P value
STAD	B Cell	Deep Deletion	0.51849727
STAD	B Cell	Arm-level Deletion	0.01064684
STAD	B Cell	Diploid/Normal	1
STAD	B Cell	Arm-level Gain	0.22262003
STAD	B Cell	High Amplication	1
STAD	CD8+ T Cell	Deep Deletion	0.21722101
STAD	CD8+ T Cell	Arm-level Deletion	0.00358451
STAD	CD8+ T Cell	Diploid/Normal	1
STAD	CD8+ T Cell	Arm-level Gain	0.00282367
STAD	CD8+ T Cell	High Amplication	1
STAD	CD4+ T Cell	Deep Deletion	0.45450308
STAD	CD4+ T Cell	Arm-level Deletion	0.00141845
STAD	CD4+ T Cell	Diploid/Normal	1
STAD	CD4+ T Cell	Arm-level Gain	0.01199774
STAD	CD4+ T Cell	High Amplication	1
STAD	Macrophage	Deep Deletion	0.23318168
STAD	Macrophage	Arm-level Deletion	0.00456658
STAD	Macrophage	Diploid/Normal	1
STAD	Macrophage	Arm-level Gain	0.0007265
STAD	Macrophage	High Amplication	1
STAD	Neutrophil	Deep Deletion	0.28499674
STAD	Neutrophil	Arm-level Deletion	4.14E-05
STAD	Neutrophil	Diploid/Normal	1
STAD	Neutrophil	Arm-level Gain	0.0389077
STAD	Neutrophil	High Amplication	1
STAD	Dendritic Cell	Deep Deletion	0.14183773
STAD	Dendritic Cell	Arm-level Deletion	1.55E-05
STAD	Dendritic Cell	Diploid/Normal	1
STAD	Dendritic Cell	Arm-level Gain	0.01030807
STAD	Dendritic Cell	High Amplication	1
