# Supplementary material for: POC1A acts as a promising prognostic biomarker associated with high tumor immune cell infiltration in gastric cancer
Source: Aging (Albany NY). 2020 Oct 14;12(19):18982–9011. doi: 10.18632/aging.103624 (PMC7732308; doi:10.18632/aging.103624)
Supplement: Supplementary Table 11 [file aging-12-103624-s006..docx]

**Supplementary Table 11** Correlation analysis between POC1A and relate genes and markers of immune cells in TIMER

|  |  | POC1A | | | |
| --- | --- | --- | --- | --- | --- |
|  |  | None | | Purity | |
| Immune Cells | Signature | Correlation | pvalue | Correlation | pvalue |
| CD8+ T Cell | ADRM1 | 0.373125 | *** | 0.3909401 | 0.3909401 |
|  | AHSA1 | 0.43507 | *** | 0.42643187 | 0.42643187 |
|  | C1GALT1C1 | 0.130606 | *** | 0.14076304 | 0.14076304 |
|  | CCT6B | -0.19879 | *** | -0.21777 | *** |
|  | CD37 | -0.29001 | *** | -0.25611 | *** |
|  | CD69 | -0.27747 | *** | -0.26032 | *** |
|  | CETN3 | 0.0641113 | 1.92E-01 | 0.06763487 | 0.06763487 |
|  | CSE1L | 0.387198 | *** | 0.39136554 | 0.39136554 |
|  | GEMIN6 | 0.277099 | *** | 0.28627776 | 0.28627776 |
|  | GNLY | 0.167395 | *** | 0.22877818 | 0.22877818 |
|  | GPT2 | 0.244444 | *** | 0.23997135 | 0.23997135 |
|  | GZMA | 0.02892658 | 5.57E-01 | 0.06023151 | 0.06023151 |
|  | GZMH | 0.01129568 | 8.18E-01 | 0.03900009 | 0.03900009 |
|  | GZMK | -0.23115 | *** | -0.21812 | *** |
|  | IL2RB | -0.0041269 | 9.33E-01 | 0.0387317 | 0.0387317 |
|  | MPZL1 | -0.16239 | *** | -0.15983 | *** |
|  | NKG7 | 0.02028683 | 6.80E-01 | 0.05489291 | 0.05489291 |
|  | PIK3IP1 | -0.48279 | *** | -0.48841 | *** |
|  | PTRH2 | 0.341733 | *** | 0.3413224 | 0.3413224 |
|  | TIMM13 | 0.44959 | *** | 0.45826988 | 0.45826988 |
| CD4+ T Cell | AIM2 | 0.03216209 | 5.13E-01 | 0.07498453 | 0.07498453 |
|  | BIRC3 | 0.00597356 | 9.03E-01 | 0.0313427 | 0.0313427 |
|  | BRIP1 | 0.413698 | *** | 0.39830676 | 0.39830676 |
|  | CCL20 | 0.266561 | *** | 0.29841181 | 0.29841181 |
|  | CCL4 | 0.0626182 | 2.03E-01 | 0.10716258 | 0.10716258 |
|  | CCNB1 | 0.656589 | *** | 0.66076337 | 0.66076337 |
|  | CCR7 | -0.26749 | *** | -0.23633 | *** |
|  | DUSP2 | 0.286974 | *** | 0.29908647 | 0.29908647 |
|  | ESCO2 | 0.54926 | *** | 0.54892367 | 0.54892367 |
|  | ETS1 | -0.34366 | *** | -0.32448 | *** |
|  | EXO1 | 0.57025 | *** | 0.56317089 | 0.56317089 |
|  | EXOC6 | 0.07712892 | 1.17E-01 | 0.07000142 | 0.07000142 |
|  | IARS | 0.203092 | *** | 0.19999438 | 0.19999438 |
|  | ITK | -0.21281 | *** | -0.18624 | *** |
|  | KIF11 | 0.604998 | *** | 0.59698932 | 0.59698932 |
|  | KNTC1 | 0.340199 | *** | 0.3376118 | 0.3376118 |
|  | NUF2 | 0.525198 | *** | 0.52293946 | 0.52293946 |
|  | PRC1 | 0.628303 | *** | 0.61636267 | 0.61636267 |
|  | PSAT1 | 0.40415 | *** | 0.42656844 | 0.42656844 |
|  | RGS1 | -0.22565 | *** | -0.20473 | *** |
|  | RTKN2 | 0.330974 | *** | 0.32294647 | 0.32294647 |
|  | SAMSN1 | -0.21461 | *** | -0.17802 | *** |
|  | SELL | -0.27724 | *** | -0.24321 | *** |
|  | TRAT1 | -0.21006 | *** | -0.18587 | *** |
| B cell | ADAM28 | -0.19393 | *** | -0.17428 | *** |
|  | CD180 | -0.24709 | *** | -0.22602 | *** |
|  | CD79B | -0.25708 | *** | -0.23881 | *** |
|  | BLK | -0.26365 | *** | -0.2504 | *** |
|  | CD19 | -0.14485 | *** | -0.12875 | *** |
|  | MS4A1 | -0.27675 | *** | -0.25968 | *** |
|  | GNG7 | -0.42846 | *** | -0.41975 | *** |
|  | MICAL3 | -0.18515 | *** | -0.18819 | *** |
|  | SPIB | -0.27056 | *** | -0.26165 | *** |
|  | HLA-DOB | -0.18175 | *** | -0.14557 | *** |
|  | PNOC | -0.149 | *** | -0.12635 | *** |
|  | FCRL2 | -0.20068 | *** | -0.18147 | *** |
|  | BACH2 | -0.45265 | *** | -0.43786 | *** |
|  | CR2 | -0.22813 | *** | -0.21698 | *** |
|  | TCL1A | -0.17216 | *** | -0.15398 | *** |
|  | ARHGAP25 | -0.24492 | *** | -0.22347 | *** |
|  | CCL21 | -0.27626 | *** | -0.26735 | *** |
|  | CD27 | -0.13461 | *** | -0.10647 | *** |
|  | CLEC17A | -0.24898 | *** | -0.22421 | *** |
|  | CLEC9A | -0.30492 | *** | -0.30182 | *** |
|  | CLECL1 | -0.29742 | *** | -0.27237 | *** |
| Macrophage | AIF1 | -0.22066 | *** | -0.18727 | *** |
|  | CCL1 | 0.07290944 | 1.38E-01 | 0.10340946 | 0.10340946 |
|  | CCL14 | -0.50423 | *** | -0.5023 | *** |
|  | CCL23 | -0.3079 | *** | -0.31364 | *** |
|  | CD300LB | -0.33192 | *** | -0.30479 | *** |
|  | CNR1 | -0.42201 | *** | -0.42276 | *** |
|  | CNR2 | -0.27334 | *** | -0.26533 | *** |
|  | EIF4A1 | 0.426473 | *** | 0.42556633 | 0.42556633 |
|  | FPR2 | 0.07925868 | 1.07E-01 | 0.14014851 | 0.14014851 |
|  | FRAT2 | 0.391495 | *** | 0.37841235 | 0.37841235 |
|  | GPR27 | -0.32089 | *** | -0.30845 | *** |
|  | GPR77 | -0.14829 | *** | -0.12616 | *** |
|  | RNASE2 | -0.1569 | *** | -0.12112 | *** |
|  | MS4A2 | -0.38836 | *** | -0.37777 | *** |
|  | BASP1 | -0.28608 | *** | -0.24563 | *** |
|  | HK3 | 0.152239 | *** | 0.20149768 | 0.20149768 |
|  | VNN1 | 0.01519875 | 7.57E-01 | 0.02529385 | 0.02529385 |
|  | FES | -0.20116 | *** | -0.19334 | *** |
|  | FAM198B | -0.46471 | *** | -0.44817 | *** |
|  | HNMT | -0.34275 | *** | -0.33416 | *** |
|  | SLC15A3 | -0.015894 | 7.47E-01 | 0.025794 | 0.025794 |
|  | CD4 | -0.17981 | *** | -0.14767 | *** |
|  | TXNDC3 | -0.20481 | *** | -0.17221 | *** |
|  | FRMD4A | -0.43337 | *** | -0.41 | *** |
|  | CRYBB1 | -0.28452 | *** | -0.28173 | *** |
|  | HRH1 | -0.13476 | *** | -0.09877 | *** |
| Neutrophil | CREB5 | -0.45899 | *** | -0.46073 | *** |
|  | CDA | -0.0073181 | 8.82E-01 | 0.0227102 | 0.0227102 |
|  | CHST15 | -0.39247 | *** | -0.37146 | *** |
|  | S100A12 | -0.0051594 | 9.17E-01 | 0.03239503 | 0.03239503 |
|  | CASP5 | 0.291204 | *** | 0.32308124 | 0.32308124 |
|  | MMP25 | 0.06518079 | 1.85E-01 | 0.1373493 | 0.1373493 |
|  | HAL | 0.0288691 | 5.58E-01 | 0.04514117 | 0.04514117 |
|  | C1ORF183 | -0.31683 | *** | -0.3171 | *** |
|  | FFAR2 | 0.16039 | *** | 0.2021298 | 0.2021298 |
|  | MAK | -0.19744 | *** | -0.1872 | *** |
|  | CXCR1 | 0.05946341 | 2.27E-01 | 0.09636104 | 0.09636104 |
|  | STEAP4 | -0.4611 | *** | -0.45697 | *** |
|  | BTNL8 | 0.01214676 | 8.05E-01 | 0.01663801 | 0.01663801 |
|  | CXCR2 | 0.00662248 | 8.93E-01 | 0.05484517 | 0.05484517 |
|  | VNN3 | 0.08595013 | 8.03E-02 | 0.10182358 | 0.10182358 |
|  |  |  |  |  |  |
